# 40 Hz light stimulation restores early brain dynamics alterations and associative memory in Alzheimer’s disease model mice

**DOI:** 10.1162/IMAG.a.70

**Published:** 2025-07-14

**Authors:** Matthieu Aguilera, Chantal Mathis, Karin Herbeaux, Amine Isik, Davide Faranda, Demian Battaglia, Romain Goutagny

**Affiliations:** Université de Strasbourg, CNRS, Laboratoire de Neurosciences Cognitives et Adaptatives (LNCA), UMR 7364, Strasbourg, France; Laboratoire des Sciences du Climat et de l’Environnement, UMR 8212 CEA-CNRS-UVSQ, Université Paris-Saclay & IPSL, CEA Saclay l’Orme des Merisiers, Gif-sur-Yvette, France

**Keywords:** Alzheimer’s disease, EEG, dynamics, gamma stimulation, GENUS

## Abstract

Visual gamma entrainment using sensory stimuli (vGENUS) is a promising non-invasive therapeutic approach for Alzheimer’s disease (AD), showing efficacy in improving memory function. However, its mechanisms of action remain poorly understood. Using young App^NL-F^/MAPT double knock-in (dKI) mice, a model of early AD, we examined brain dynamics alterations before amyloid plaque onset. High-density EEG recordings and metrics from fields outside neuroscience were used to assess brain dynamics fluidity—a measure of the brain’s ability to transition between activity states. We revealed that dKI mice exhibit early, awake state-specific reductions in brain dynamics fluidity associated with cognitive deficits in complex memory tasks. Daily vGENUS sessions over 2 weeks restored brain dynamics fluidity and rescued memory deficits in dKI mice. Importantly, these effects built up during the stimulation protocol and persisted after stimulation ended, suggesting long-term modulation of brain function. Based on these results, we propose a “brain dynamics repair” mechanism for vGENUS that goes beyond current amyloid-centric hypotheses. This dual insight—that brain dynamics are both a target for repair and a potential diagnostic tool—provides new perspectives on early Alzheimer’s disease pathophysiology.

## 1 Introduction

Alzheimer’s disease (AD) represents the leading cause of dementia worldwide, characterized by progressive memory loss and cognitive decline. Despite decades of research, effective treatments and early diagnostic tools remain elusive, a challenge attributable in part to the field’s prolonged focus over the three last decades on the amyloid hypothesis, which posits that Amyloid-beta (Aβ) accumulation drives cognitive deficits (Hardy & Higgins, 1992; Selkoe, 1991; Selkoe & Hardy, 2016). However, mounting evidence challenges this framework: memory deficits often precede amyloid plaque formation (Jacobsen et al., 2006; Van Dam et al., 2003), can occur independently of Aβ (Hamm et al., 2017), and amyloid plaques sometimes appear in asymptomatic individuals (Iacono et al., 2014; Rowe et al., 2010). These discrepancies, combined with repeated failures of Aβ-targeted therapies, failing to reverse AD symptoms and displaying severe side effects as reduction of cognitive ability, vasogenic edema, and increased skin cancer, inflammation and infections (Cummings et al., 2014; Doody et al., 2013, 2014; Orgogozo et al., 2003; Wilcock & Colton, 2009), underscore the urgent need for alternative early biomarkers that capture broader aspects of disease progression.

One such early marker could be alterations in brain system dynamics which co-occur with or even precede memory impairments. Oscillatory disruptions, such as impaired rhythmic coordination and altered spectral power, are widely reported in AD (Baker et al., 2008; Claus et al., 1998; Dauwels et al., 2011; Moretti et al., 2009) and, in some cases, precede Aβ deposition (Goutagny et al., 2013; Hamm et al., 2017). Broader disturbances in global brain dynamics have also been observed (Arbabyazd et al., 2023; Lian et al., 2021; Tait et al., 2020). There is a fundamental reason why we care about brain dynamics. Several studies have shown that cognitive efficiency across different domains is closely linked to the brain’s ability to flexibly transition between alternative coordination states—more so than the occurrence of any specific state (Bassett et al., 2011; Battaglia et al., 2020; Braun et al., 2015). This finding is particularly compelling, as fundamental theories of computation suggest that a necessary condition for a system to support effective computation is its capacity to update its internal states quickly and in a structured manner (Turing, 1937). If efficient computation depends on “good dynamics”, then impaired or “bad” dynamics could underlie difficulties in information processing. To detect such issues, it is essential to utilize analytical approaches that avoid relying on a priori assumptions—methods that do not depend on averaging across predefined frequency bands, heavily processed indirect connectivity measures, or partially arbitrary clustering algorithms. These transformations, while useful, may obscure subtle yet functionally significant features of the system’s intrinsic, time-evolving dynamics. To overcome these limitations, we introduce a data-driven method that remains as faithful as possible to the original continuous time activity time series. Specifically, we investigate how the system’s dynamical flow either tends to dwell around specific transient configurations or, alternatively, transitions smoothly across varying configurations. While conventional metrics of dynamic stability from nonlinear dynamics are difficult to estimate without knowledge of governing equations (Wolf et al., 1985), limiting their applicability in experimental neuroscience, we apply a time-resolved, model-free metric capitalizing on a recent application of extreme value statistics results to dynamical systems theory (Faranda et al., 2017). This measure, which we term *dynamics fluidity*, tracks temporal clustering of system’s configurations in dynamical phase space without prior assumptions about the system’s structure. Originally developed for atmospheric systems, dynamics fluidity offers a novel perspective on early alterations in brain dynamics that may underlie cognitive dysfunction in AD.

If early alterations in global brain dynamics are a hallmark of AD, then interventions capable of restoring these dynamics may represent a promising therapeutic strategy. One such intervention is Gamma ENtrainment Using Sensory stimuli (GENUS; Singer et al., 2018), which has demonstrated memory benefits in AD mouse models (Adaikkan et al., 2019; Martorell et al., 2019) and in patients with mild probable AD (Chan et al., 2022). Proposed mechanisms include reduced neurodegeneration and improved memory via decreased amyloid plaque burden, primarily through microglial activation, glial responses (Adaikkan et al., 2019; Martorell et al., 2019), or enhanced clearance pathways (Murdock et al., 2024).

However, recent studies have questioned the specificity and efficacy of these mechanisms. Some report limited propagation of 40 Hz entrainment beyond primary sensory cortices and minimal engagement of memory-relevant regions such as hippocampal CA1 (Schneider et al., 2023), while others question whether genuine gamma oscillations are being entrained at all (Soula et al., 2023). Moreover, the impact of GENUS on amyloid load remains debated (Soula et al., 2023; Yang & Lai, 2023). Despite these uncertainties, most discussions remain anchored in the amyloid-centric framework.

Here, we propose an alternative hypothesis: that GENUS may restore memory function not through 40 Hz-specific or amyloid-related effects, but by re-establishing altered global brain network dynamics. This perspective is supported by emerging evidence that GENUS influences brain activity patterns even outside of AD pathology (Blanpain et al., 2024; Zhang et al., 2021), pointing to broader impacts on neural function.

To test this hypothesis, we conducted high-density EEG (hdEEG) recordings in a preclinical AD mouse model (App^NL-F^/MAPT double knock-in mice; Saito et al., 2019; dKI, n = 8, both sexes) and control littermates (n = 8, both sexes) during memory task performance. Our previous work (Borcuk et al., 2022) demonstrated that at 4 months, dKI mice maintain normal performance in simple tasks (e.g., short-term Novel Object Recognition or Object Location) but exhibit specific and subtle deficits in more complex associative memory tasks such as Object–Place association. In this study, we assessed both memory performance and large-scale brain dynamics during Object–Place association and long-term Novel Object Recognition tasks, before and after a 2-week course of daily 1-hour visual GENUS (vGENUS).

## 2 Methods

### 2.1 Animals and surgery

#### 2.1.1 Animals and ethics

Double Knock-in App^NL-F^/MAPT (dKI) and Wild-Type (WT) mice were obtained as described in Borcuk et al. (2022). For EEG–behavior experiments, 8 WT and 8 dKI mice were housed in individual cages post-surgery. For behavior-only experiments, 35 WT and 37 dKI 4 months old mice were housed in individual cages. All animals were under a 12-hour light/dark normal cycle (light-on during daytime and off during nighttime) with food and water present *ad libitum*. Both sexes were balanced in population to get closer to real population representation without studying sex effect. All experimental protocols agreed with the European Committee Council directive (2010/63/UE) and were approved by the French Ministry of Research (APAFIS#28839-2021010509459441).

#### 2.1.2 Surgery

Surgeries were performed at 3 months old. Animals were anesthetized with isoflurane (IsoFlo, Zoetis) during the entire surgery (4% for induction then maintained at 1.5%). Local anesthesia was performed on incision site by bupivacaïne and lidocaïne (Lurocaine, Vetoquinol) injection prior to incision. Post-surgery analgesia was provided via sub-cutaneous Metacam injection. EEG surface grid (H32 mouse EEG grid, Neuronexus, Ann Arbor, USA) was placed on the skull aligning the skull bregma with the grid landmark. The grid is fixed to the skull by applying saline and letting it dry. One screw was inserted above the right cerebellum to serve as ground, and another one, placed rostral to the grid, was used as fixation support for the implant. The reference used was the internal reference of the electrode. The implant was secured for long-term use using dental glue (Super-bond, Sun Medical) and dental cement (Paladur).

### 2.2 Mice perfusion and immunochemistry

Mice were deeply anesthetized with an intraperitoneal injection of ketamine (200 mg/kg) and xylazine (30 mg/kg) and then transcardially perfused with 0.1% heparin in 0.1 M phosphate-buffered saline (PBS), followed by 4% paraformaldehyde (PFA) in 0.1 M phosphate buffer (PB; pH 7.4, 4°C). Brains were post-fixed in PFA for 24 hours, cryoprotected in 20% sucrose in PB for 48 hours, and subsequently stored at -80°C. Coronal sections (40 µm) were obtained using a cryostat, covering the region from Bregma +2.58 to Bregma -5.88. Labeling was performed on one section every 160 µm, yielding approximately 62 sections per animal. Sections were processed at room temperature with agitation at 260 rpm as follows: 3 washes with PBS, followed by a 15-minute incubation in 70% formic acid, another 3 PBS washes, a 30-minute incubation in methanol with 0.3% H_2_O_2_, a 15-minute wash in ultrapure water, and 2 additional PBS washes. Sections were blocked for 1 hour in 5% normal horse serum (NHS) diluted in PBS containing 0.5% Triton X-100, followed by an 18-hour incubation at 4°C with mouse anti-6E10 antibody (1:1000 in 2% NHS, BioLegend #803001). After three PBS washes, sections were incubated for 2 hours with a biotinylated horse anti-mouse antibody (1:500 in PBS containing 0.5% Triton X-100, Vector Laboratories, #BA-2001-.5), followed by three PBS washes. Sections were then incubated for 30 minutes in an avidin–biotin solution (Vector Laboratories), washed twice in PBS, and finally in PB-Tris. Detection was performed using a 15-minute incubation in 3,3-diaminobenzidine (Vector Laboratories). Sections were mounted onto gelatin-coated slides, air dried for 24 hours, dehydrated through a graded series of alcohol baths (70%, 90%, 95%, 100%, 100%), cleared with Clearify (American MasterTech Scientific), and fixed on microscopic slides with Diamount (Diapath S.P.A). Slides were then dried for 48 hours in the dark. Whole-section images were acquired at 20x magnification using a Hamamatsu NanoZoomer S60 digital slide scanner (Hamamatsu Photonics K.K., Japan). Amyloid plaques were counted using the cell counter tool in ndpView2 software (Hamamatsu Photonics K.K., Japan). Plaques were identified based on their shape and the density of 6E10 labeling relative to the background. Specificity of labeling was confirmed by including a negative control in which the primary antibody (6E10) was omitted. As a positive control, sections from two 12-month-old dKI mice were processed and analyzed using the same protocol at the same time.

### 2.3 Behavior

#### 2.3.1 Apparatus

Behavioral tasks are conducted in a 55 cm x 55 cm open field with black wall and a white ground with a grid pattern.

#### 2.3.2 Habituation

Two weeks after surgery, animals were habituated to the experimental apparatus. Transportation box was progressively presented over a week for the transport habituation. A recording cable was plugged several hours per day for 2 days to the head-mounted pre-amplifier so the mice could get used to its presence and weight. During the 3 days prior the behavioral task, an Open Field habituation consisting in 10 minutes exploration of an empty Open Field and 2 object habituations consisting in two 10-minute sessions with a single object in the open field were performed.

#### 2.3.3 Novel object recognition task

At 4 months old and after vGENUS protocol, after the previously described habituation, mice perform a Novel Object Recognition (NOR) task. The mouse explores the open field where 2 similar objects are placed during a 10-minute sampling phase, and 24 hours after, realizing a 10-minute test phase consisting in an exploration of the open field where one of the previous objects is replaced by a new one. Objects exploration time is measured during the sampling and the test phase. To avoid place preference, the object replaced is the one that was the less explored during the sampling phase.

#### 2.3.4 Object in place task

At 4 months old and after vGENUS protocol, after the previously described NOR task, mice perform an Object in Place task. The mouse explores the open field where 2 different objects are placed during a 10-minute sampling phase, then wait a 5-minute inter trial interval in the home cage, before a second 10-minute test phase consisting in an exploration of the open field where one of the previous objects is replaced by the copy of the other. Here neither the environment nor the object is new during the test phase but only the association between the specific object and its place in the environment. Objects exploration time is measured during the sampling and the test phases. To avoid place preference, the object replaced is the one that was the less explored during the sampling phase.

#### 2.3.5 Memory performances

To assess memory performances, a memory index (MI) is computed from object exploration times during test as follows:

$$MI=\;\frac{New\;Object\;Exploration\;Time\;-\;Familiar\;Object\;\;Exploration\;Time\;}{New\;Object\;Exploration\;Time\;+\;Familiar\;Object\;\;Exploration\;Time}$$

#### 2.3.6 Sleep recordings

After the sampling phase of the NOR, mice are recorded in their home cage for a minimum of 6 hours during light phase to record first post-learning sleep episodes.

#### 2.3.7 Video recordings

Behavioral tasks are recorded with a camera placed over the open field and filming it from a top view at a constant 25 frame per second rate.

#### 2.3.8 Video tracking

Video tracking of the animal is performed *a posteriori* using DeepLabCut (Mathis et al., 2018). The animal is tracked at eight distinct points forming its skeletal structure: the nose, both ears, the neck, the midpoint of the body, the tail base, the middle of the tail, and the tail end. X and Y coordinates for each point are extracted for every frame of the video.

#### 2.3.9 Preprocessing of DeepLabCut data

Since the apparatus can shift slightly between videos, normalizing the coordinates obtained from DeepLabCut is crucial for comparing different recordings. For each video, the coordinates of the four corners of the Open Field are manually identified. Using these, we calculate the vector *OA_OC*, which represents the offset between the origin of the Open Field reference frame and the camera’s original referential, along with the angle θ (i.e., the angle between the top wall of the Open Field and the upper border of the camera’s field of view). The *OA_OC* vector allows for centering the X and Y coordinates of each body parts ζ within the new camera referential as follows:

$$\left[\begin{array}{c} X_{\zeta OpenField}\\ Y_{\zeta OpenField} \end{array}\right]=\left[\begin{array}{c} X_{\zeta Camera}\\ Y_{\zeta Camera} \end{array}\right]+OA\_OC.$$

Then θ angle allows the computation of the rotation matrix as follows:

$$RM=\;\left[\begin{array}{cc} cos\theta & -sin\theta \\ sin\theta & cos\theta \end{array}\right].$$

The rotation matrix is then used to rotate the coordinates to align with the Open Field referential as follows:

$$\left[\begin{array}{c} X_{\zeta realigned}\\ Y_{\zeta realigned} \end{array}\right]=RM\;\;\cdot \;\;\left[\begin{array}{c} X_{\zeta OpenField}\\ Y_{\zeta OpenField} \end{array}\right].$$

Thus, body parts coordinates are now represented in a referential where the bottom wall of the Open Field aligns with the X-axis and the left wall with the Y-axis. The final step consists in normalizing the distances so the length of each Open Field wall spans from 0 to 1. To do so, each X coordinate of the body parts is divided by the norm of the vector representing the bottom wall and each Y coordinate is divided by the norm of the vector representing the left wall.

#### 2.3.10 Barycenter extraction

For several measures where precise body parts are unnecessary and only the animal’s overall position is required, we use a reference point called the barycenter. The barycenter is obtained by averaging the coordinates of the neck and body for each frame.

#### 2.3.11 Speed computation

To compute speed, the instantaneous speed between frames is computed based on barycenter coordinates. First the vector of the barycenter displacement (BD) between the two frames is computed in complex numbers as follows:

$$\begin{array}{l} BD\left(t\right)=\left(BarycenterX\left(t\right)-BarycenterX\left(t-1\right)\right)\\ \;\;\;\;\;\;\;\;\;\;\;\;\;\;\;\;\;\;\;\;\;\times i\left(BarycenterY\left(t\right)-BarycenterY\left(t-1\right)\right) \end{array}$$

From this, the instantaneous speed is computed as the absolute of BD. The average speed is then used for speed comparisons.

#### 2.3.12 Posture extraction

For each point ζ of the skeleton, the X and Y coordinates are normalized by the barycenter coordinates effectively centering them on the barycenter as follows:

$$\left[\begin{array}{c} X_{\zeta centered}\\ Y_{\zeta centered} \end{array}\right]=\left[\begin{array}{c} X_{\zeta }-X_{Barycenter}\\ Y_{\zeta }-Y_{Barycenter} \end{array}\right].$$

Next, a rotation correction is applied to eliminate the effect of the animal’s orientation in space, ensuring a consistent desired orientation. The angle φ is defined as the rotation angle between the barycenter and the realigned “neck” position. Each skeletal point ζ is then realigned using the following transformation:

$$\begin{array}{l} Coordinates_{\zeta realigned}=(X_{\zeta centered}+iY_{\zeta centered})e^{i(\frac{\pi }{2}\varphi )}\\ \;\;\;\;\;\;\;\;\;\;\;\;\;\;\;X_{\zeta realigned}=ℜ(Coordinates_{\zeta realigned})\\ \;\;\;\;\;\;\;\;\;\;\;\;\;\;\;\;\;Y_{\zeta realigned}=ℌ(Coordinates_{\zeta realigned}). \end{array}$$

#### 2.3.13 Head movements

Head movements are determined for each frame by taking the norm of the vector of head points coordinates (nose, left ear, right ear) between time *t* and *t-1* as follows:

$$Head\;Movement\;\left(t\right)=\left\|\begin{array}{c} X_{\left[Nose,\;EarLeft,EarRight\right]realigned}\left(t\right)-X_{\left[Nose,\;EarLeft,EarRight\right]realigned}\left(t-1\right)\\ Y_{\left[Nose,\;EarLeft,EarRight\right]realigned}\left(t\right)-Y_{\left[Nose,\;EarLeft,EarRight\right]realigned}\left(t-1\right). \end{array}\right\|$$

### 2.4 Visual Gamma Entrainment Stimulation (vGENUS) protocol

Visual GENUS protocol and setup were performed as described in Singer et al. (2018). The day after the performance of the OiP task, start a 15-day protocol. Each day, mice are placed in their home cages in the stimulation room with light on for a 1-hour stalling. Mice are then individually placed in stimulation cages, consisting in regular cages with three black painted walls to let transparent only the wall facing the stimulation light, without litter, food, and water access. For 1 hour, the light of the room is turned off and only a 12 V LED strip with a 4000/4500K light temperature and a 1200 lm/m (Ledkia) controlled by an Arduino is either flickering at 40 Hz or displaying continuous light as a control condition. As previous studies have shown that stimulation at other frequencies (e.g., 8 Hz, 20 Hz, or 80 Hz) does not produce beneficial effects on AD-related phenotypes (Martorell et al., 2019; Singer et al., 2018), we limited our protocol to 40 Hz stimulation. This allowed us to avoid cross-protocol interference and focus specifically on the frequency with the most consistently reported therapeutic potential in AD models. Mice implanted with EEG probes were electrophysiologically recorded during the stimulation. After the stimulation, mice are back in the home cage for a 1-hour stalling in the stimulation room with the light on and the LED strip off before returning to the animal facility or doing behavioral task.

### 2.5 Electrophysiological recordings and analysis

#### 2.5.1 Recording and preprocessing

The electrophysiological activity was recorded with an Intan recording controller (RHD Recording Controller, Intan Technologies, USA). The signals were amplified 200 x, recorded whole-band (0.1 Hz–15 kHz) and digitized at 1 kHz. Qualitative rejection of faulty channels was conducted, any animals presenting more than five faulty channels were excluded from the study. EEG signals were then lowpass filtered at 100 Hz and artifact-related activity was removed via Independent Component Analysis (ICA). The signals were then coarse grained using a 40 ms time window to reduce the computational load of subsequent analyses and to match the frame rate of the behavioral video recordings, enabling time-resolved analysis of the relationship between brain dynamics and fine-grained behavioral features. We acknowledge, therefore, that contribution of higher frequencies may be underestimated in all subsequent analysis.

#### 2.5.2 Spectral analysis

All spectral analyses were conducted on non-coarse-grained signals using Matlab Chronux toolbox *mtspecgram* function. Relative power was averaged across electrode groups positioned over specific cortical areas (Prefrontal Cortex: PFC; Motor Cortex: MOC; Somatosensory Cortex: SSC; Parietal Cortex: PAR; Retrosplenial Cortex: RSC; Visual Cortex: VIS) based on the Allen Institute mouse cortical parcellation. This grouping reflects electrode positioning rather than source-localized signals as all signals were not source localized.

##### 2.5.2.1 Frequency band power

Spectrograms were computed for behavioral task and sleep recordings using a 10-second time window with a 5-second overlap and 5 tapers with a 3-second time bandwidth, providing a time-resolved representation of frequency and power content. The power spectrum was obtained by averaging the spectrogram over the entire recording duration. To normalize, each point in the power spectrum was divided by the total power across all frequencies. Relative frequency band power was then calculated as the area under the curve for specific frequency ranges: delta (0.5–4 Hz), theta (4–12 Hz), and gamma (30–80 Hz).

##### 2.5.2.2 40 Hz power

Spectrograms were computed for 10 minutes recordings during vGENUS stimulation using a 60-second time window with a 30-second overlap (longer time window was used for better frequency resolution of the 40 Hz peak) and 5 tapers with a 3-second time bandwidth, providing a time-resolved representation of frequency and power content. The power spectrum was obtained by averaging the spectrogram over the entire recording duration. Relative 40 Hz power was obtained by dividing the 40 Hz power by the total power across all frequencies.

#### 2.5.3 Microstates extraction with k-mean clustering

Microstates were extracted independently for each animal and each trial to favorize individual characterization. To extract microstates sequences in an unsupervised way, k-mean clustering using native Matlab function with correlation distance was performed on the coarse-grained EEG time series for a wide range of cluster extraction (3 to 8).

#### 2.5.4 Microstates extraction with global field power peaks

To extract EEG microstates through a procedure closer to the human EEG literature (Michel & Koenig, 2018), global field power was computed as the variance of the coarse-grained EEG of all channels at each time points. Peaks of GFP were determined using a minimum peak prominence of 0.5. EEG at GFP peaks was then clustered using k-mean clustering with native Matlab function with correlation distance and centroid were extracted. Microstates sequences were constructed by applying at each time point of the coarse-grained EEG, the centroid showing the highest Pearson correlation.

#### 2.5.5 Microstates fluidity

To study switching across microstates, transition probability matrices were computed as the probability to switch from a microstate visited at time *t* to a different microstate at times *t*+1. Diagonal entries in this transition matrix correspond to the probability of remaining in the currently visited microstate without switching, which is proportional to the average dwell time within the considered microstate. The average of diagonal transition matrix entities was thus a measure of microstates stability as it represents the average probability to remain in the same state without switching. We then defined the inverse of this microstate stability as microstates fluidity as follow:

$$Microstates\;Fluidity=\;\frac{1}{Mean\;of\;transition\;matrix\;diagonal}$$

Such a quantity decreases if dwell times increase and time to the next microstate switching is increased.

#### 2.5.6 Microstates sequence complexity

Microstates sequence complexity is computed following Clawson et al. (2019), through a minimum description length approach. Following ideas proposed by Kolmogorov and Chaitin (for review see Chaitin, 1987), the shortest the symbolic sequence can be described in a suitable lossless-compressed format, the less complex it is. Each microstates sequence is considered as a sequence of symbolic labels corresponding to the microstate visited at each time. Let suppose that ***A***, ***B***, ***C**…* are the labels of different microstates. Their set forms the dictionary ∆. An original description of the sequence can be given by listing all the individual positions in the sequence where a given label is used, for example, ***A** a_1_ a_2_ a_3_ …**B** b_1_ b_2_ b_3_ …**C** c_1_ c_2_ c_3…_* where *a_i_, b_i_, c_i_* …are positions where the labels ***A, B, C***…, respectively, appear in the sequence. The length of the original description is thus given by

$$L_{orig}=\;\left|\Delta \right|+N_{words},$$

where |*∆*| is the size of the dictionary and *N_words_* is the number of labels in the symbolic stream. If there are repetitions of consecutive symbols associated with permanence within the same microstate for a certain epoch of longer duration than a single time step, a potentially compressed description of the same sequence can be obtained using a different encoding, ***A** l_1_ s_1_ l_2_ s_2_…**B** l_1_ s_1_ l_2_ s_2_…**C** l_1_ s_1_ l_2_ s_2…,_* where the numbers *l_i_* and *s_i_* occurring alternatingly after a symbolic label, for example, ***A***, correspond, respectively, to the length *l_i_* of the *i*-th block of consecutive symbols ***A*** and to the shift of positions to find within the sequence the next (*i+1*)-th block of symbols ***A***. If the number of blocks of symbols is much smaller than the number of individual symbols in the sequence, then the length *L_comp_* of this second description will be shorter than $L_{orig}$ . The complexity of the state transitions was quantified as the ratio of the compressed length to the original length Specifically, we also applied a base 100 exponential transformation so that the obtained values follow a normal distribution and statistical testing is simplified:

$$DL_{Complexity}=100^{\frac{L_{comp}}{L_{orig}}}.$$

#### 2.5.7 Brain dynamics fluidity

What we call here dynamics fluidity corresponds to a quantity which has been called inverse persistence in the literature about the analysis of dynamical systems and, specifically, climatic time series (Faranda et al., 2017). This quantity is evaluated based on empirical multivariate and continuous-valued time series, here the 30-dimensional coarse-grained multi-channel EEG. The mathematical theory of this quantity is sophisticated, and we invite the reader to refer to the original publications for full details (Freitas et al., 2010; Lucarini et al., 2012; Moloney et al., 2019). We can, however, provide some intuition. We consider the values $u_{t,i}$ of the EEG activity of different channels *i* at time *t* as describing a trajectory ***u_t_*** in a high-dimensional space. We can then consider a system configuration ***u_0_*** visited by the system at a certain time and ask how much time is needed for the system’s trajectory leaving a neighborhood of the current configuration, that is, flowing away to far configurations that do not resemble ***u_0_***. The fluidity of the dynamics in proximity of the configuration ***u*** will be inversely reflecting the persistence time. The more persistent the configuration ***u***, the longer the previous and subsequent states of the system will resemble ***u***, and the lower will be the dynamics fluidity. To estimate fluidity from time series observations, a connection between dynamical systems theory and extreme-value statistics can be exploited. To quantify the deviation of the trajectory *u_t_* from the reference point *u_0_*, we introduce a distance measure *dist*(*u_t_   , u_0_*), using the Euclidean distance. We are interested in detecting clustering behavior, that is, “sticky” behavior where the trajectory *u_t_* spends more time than usual around the reference point *u_0_*. To highlight the short distance values in these clustering situations, we use the log distance and define the function *g*(*t;u_0 _*) = −log(*dist*(*u_t_   , u_0_*)). This function is large when the trajectory *u_t_* is in proximity to *u_0_*. Consequently, the probability of persistence or returns around *u_0_* corresponds to the probability of observing extreme values of *g*(*t*;*u_0_* ).

Requiring that a point on the orbit falls within a ball of radius e^−^*^s^* around *u_0_* is equivalent to asking that the corresponding value of the series *g*(*t*) exceeds the threshold s. Assuming independence of exceedances *g*(*t*), we obtain

$$P\left[\left(X-s\left(q\right)\right)>y\;|X\geq s\left(q\right)\right]\;≅\text{exp}\left[-\left(\frac{y}{\sigma \left({\text{u}}_{0}\right)}\right)\right],$$

where *s(q)* is a high threshold associated with a quantile *q* of the series *X* ≡ *g*(*t*). The resulting distribution is an exponential member of the Generalized Pareto Distribution (GPD) family. The parameter σ, the scale parameter of the distribution, depends on the point *u0* in phase space and, for finite time series, on *t*. If we define *M_n_* = max(*X_0_, X_1_,…,X_n−1_),* we can then write

$$P\left[M_{n}<y\right]\;≅\text{exp}\left[-\theta n\exp\left(-\frac{y}{\sigma }\right)\right],$$

where *M_n_* is the maximum of the series over *n* time steps. The details of this computation are given in Faranda et al. (2023). A metric of persistence can then be obtained as the inverse of the extremal index θ, dimensionalized by the time step of the data used, whereas σ can be linked to the dimensionality of the data.

The parameters θ, μ, and σ can be fitted practically on data using a suitable maximum likelihood estimator, by adopting a large threshold η. MATLAB code is provided in the Supporting Material for their evaluation, based on the Süveges estimator (Süveges, 2007). In our study, we set η to be equal to the 98% quantile of the distribution of *g(t;**u**_0_).* The quantity that we call here dynamics fluidity corresponds to the fitted parameter θ. The larger will be θ, the less persistent (and thus the more fluid) will be the dynamics. The case of θ = 0 corresponds notably to ***u**_0_* being a fixed point, so that the probability of remaining in its proximity after having reached it is 1. The case of θ = 1 corresponds to the probability of the return time being a Poisson distribution and thus to the absence of trajectory points clustering. Furthermore, the parameter σ provides information on the local dimension of the attractor manifold surrounding ***u**_0_* (see again Faranda et al., 2017), but we do not exploit this information in this study.

The estimated parameters, including dynamics fluidity θ, depend on the local point ${\text{u}}_{0}$ chosen as reference. Thus, fluidity analysis of a multivariate time series of EEG will yield a time series of values of fluidity, each estimated using as reference a different instantaneous observation of EEG multi-channel topography.

#### 2.5.8 EEG x behavior relationship

To assess potential relationship between EEG dynamics fluidity and animal locomotor measures as speed and head movements, we computed first mutual information between the two variables.

First, both EEG and locomotor measures were discretized into deciles using quantile binning. The sequences were then bootstrapped 100 times by either shuffling EEG and each locomotor measures keeping the temporal association between the two or independently shuffling the two time series to generate a null model. For each bootstrap iteration, mutual information between the two time series was computed as follows:

$$MI\left(i,j\right)=\;\sum_{i,j}P\left(i,j\right)log_{2}\frac{P\left(i,j\right)}{P\left(i\right)P\left(j\right)},$$

where i and j represent the decile states (1–10) of EEG and locomotor measure, respectively, *P(i,j)* is the joint probability distribution, and *P(i)* and *P(j)* are the marginal probability distributions.

The MI was then normalized by the largest entropy between the two time series:

$$H=\max\left(-\sum_{i}P\left(i\right)log_{2}P\left(i\right)\;;\;-\sum_{j}P\left(j\right)log_{2}P\left(j\right)\right).$$

The mean *MI/H* of bootstrap distribution was kept for each subject and experimental condition. The mean *MI/H* of independent bootstrap null model was used to compare bootstrap data against chance levels.

The direction of relationships between EEG fluidity and locomotor measures across experimental conditions was assessed using a bootstrap-based co-distribution analysis, designed to quantify their association while accounting for sampling variability. For each combination of task and genotype, both before and after vGENUS, EEG fluidity values were divided into deciles (based on the 0:10:100 percentile range), and the median of the locomotor measure was computed within each decile. To evaluate the statistical reliability of this relationship, a bootstrapping procedure with 200 iterations was performed. In each iteration, two types of distributions were generated: (1) a null distribution (H0), in which EEG fluidity and locomotor measure values were independently resampled with replacement, thereby disrupting any existing relationship and (2) a conditional distribution (H1), in which both variables were resampled using the same index vector, preserving their original pairing. From these resampled datasets, decile-wise medians of the locomotor measure were computed for each iteration, yielding bootstrapped estimates of the co-distribution under both hypotheses. This approach enabled the characterization of how locomotor measures varied across levels of EEG dynamics fluidity, and whether the observed relationship exceeded what would be expected under random association. The method was applied uniformly across all conditions, providing a consistent framework for comparing the relationship between EEG fluidity and locomotor behavior across tasks and genotypes, before and after vGENUS.

### 2.6 Statistical analysis

All statistics were performed using either built-in Matlab (r2024a) functions, Toolboxes, custom script, or Jamovi 2.5. For all statistical tests, the significance threshold α was fixed at 0.05.

#### 2.6.1 Behavior

**Fig. 1.**
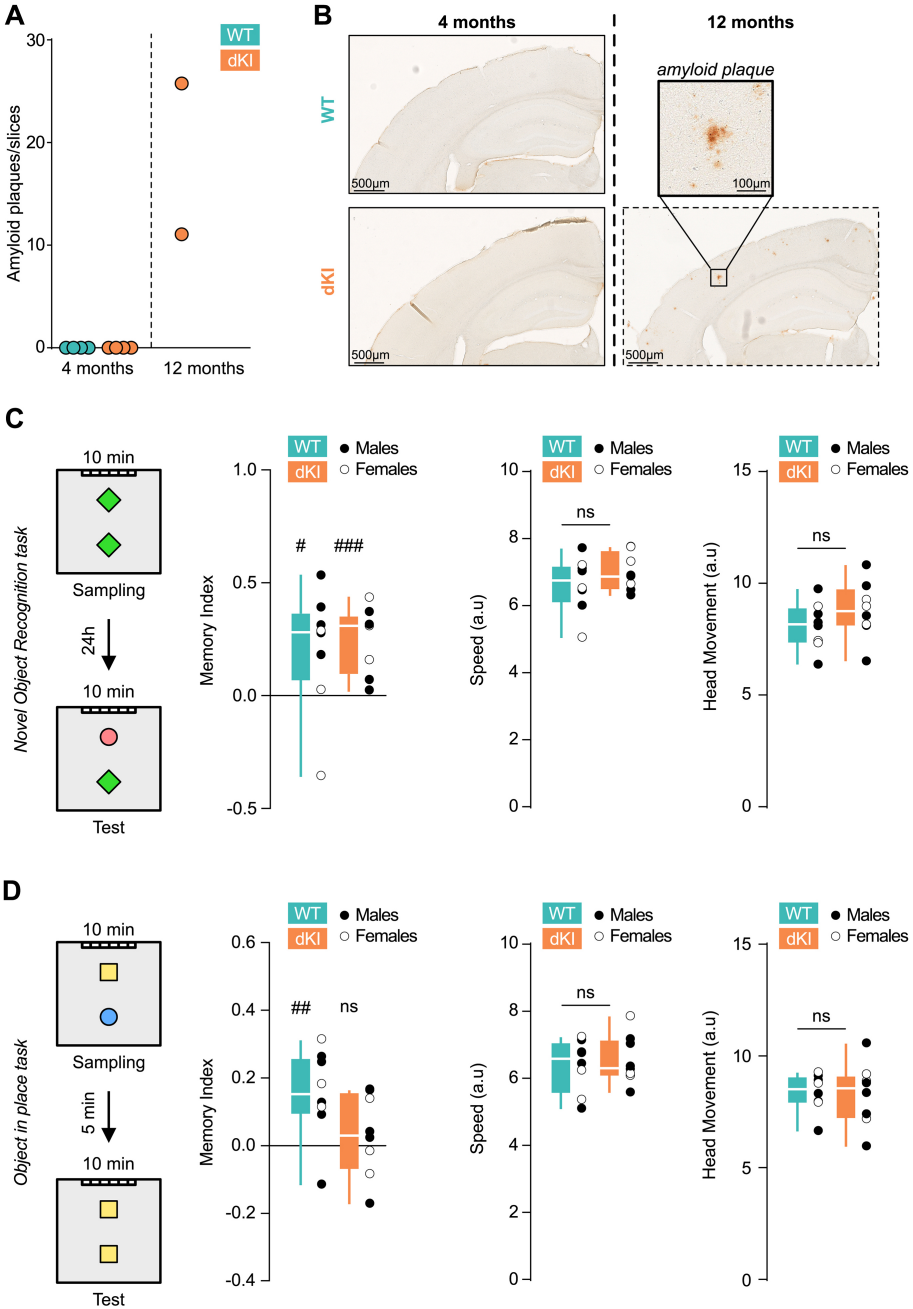
Young dKI mice display memory deficits in complex task before amyloid plaques onset. (A) Number of amyloid plaques per brain slices at 4 months (left) for WT (n = 4, blue) and dKI (n = 4, orange). We detect only 1 plaque in 262 brain slices from 4 dKI mice. As a positive control, plaques were counted for 12 months old (right) dKI (n = 2, orange). Plaques were counted over numerous frontal brain slices of several brain region plans. The total amount of plaques counted was divided by the total amount of slices for each animal. (B) Amyloid plaques immuno-staining of dorsal hippocampus at 4 months (left) for a WT (top) and a dKI (bottom) mouse and at 12 months (right) for a dKI mouse. Amyloid plaques were present in dKI mice at 12 months (top right for magnification) but not at 4 months. (C) Novel Object Recognition (NOR) task protocol (left), memory index for WT (n = 8, blue), and dKI (n = 8, orange) mice (center left), speed (center right), and head movement of the animals (right). Both WT and dKI show memory performances higher than chance levels (one-sided t-test against chance, #: p < 0.05, ##: p < 0.01, ###: p < 0.001), and no differences in speed or head movement are observed between genotypes (two-sided Wicoxon Mann–Whitney test between genotypes). Box ranges from 25 to 75 percentile and whiskers for minimum to maximum values, median is represented by white line, individual points are displayed in black for males and white for females. (D) Object-in-Place (OiP) task protocol (left), memory index for WT (n = 8, blue) and dKI (n = 8, orange) mice (center left), speed (center right), and head movement of the animals (right). dKI mice show memory performances not higher than chance levels (one-sided t-test against chance, #: p < 0.05, ##: p < 0.01, ###: p < 0.001) and almost significantly lower than WT (two sample two-sided t-test, p = 0.08). However, no differences in speed or head movement were observed between genotypes (two-sided Wicoxon Mann–Whitney test between genotypes). Box ranges from 25 to 75 percentile and whiskers for minimum to maximum values, median is represented by white line, individual points are displayed in black for males and white for females.

Memory index was analyzed using either unpaired t-test between both genotypes for each task before and after vGENUS for implanted animals displayed in Figures 1 and 5; two-way ANOVA (factor: Genotype, Task) were used on the larger non-implanted cohort before vGENUS; and repeated two-way ANOVA (non-repeated factor: Genotype; repeated factor: vGENUS) were used on the larger non-implanted cohort after vGENUS. Post hoc tests were used when necessary, using a Bonferroni correction for multiple comparison. Before and after GENUS, memory index was compared for each genotypes against chance levels using one-sided t-test testing H_1_ > 0 or memory index “above” chance levels. Exploration time was analyzed using a three-way ANOVA (factor: genotype, task, and phase (sampling/test)) before vGENUS and three-way repeated ANOVA (non-repeated factor: genotype and phase (sampling/test); repeated factor: GENUS (before/after)) after vGENUS. Post hoc tests were used when necessary, using a Bonferroni correction for multiple comparison.

#### 2.6.2 Relative spectral power

Differences in relative spectral power across cortical areas between genotypes were assessed using multiple t-tests for each region and frequency band, separately for behavior and sleep, both before and after GENUS. Multiple comparisons were corrected using Bonferroni correction. GENUS effect was tested using three-way repeated ANOVA (non-repeated factor: genotype and task or sleep stage; repeated factor: GENUS (before/after)). Differences of 40 Hz relative power were assessed through three-way repeated ANOVA (non-repeated factor: genotype and region; repeated factor: Day of GENUS (Day 1/Day 15)).

#### 2.6.3 EEG microstates

**Fig. 2.**
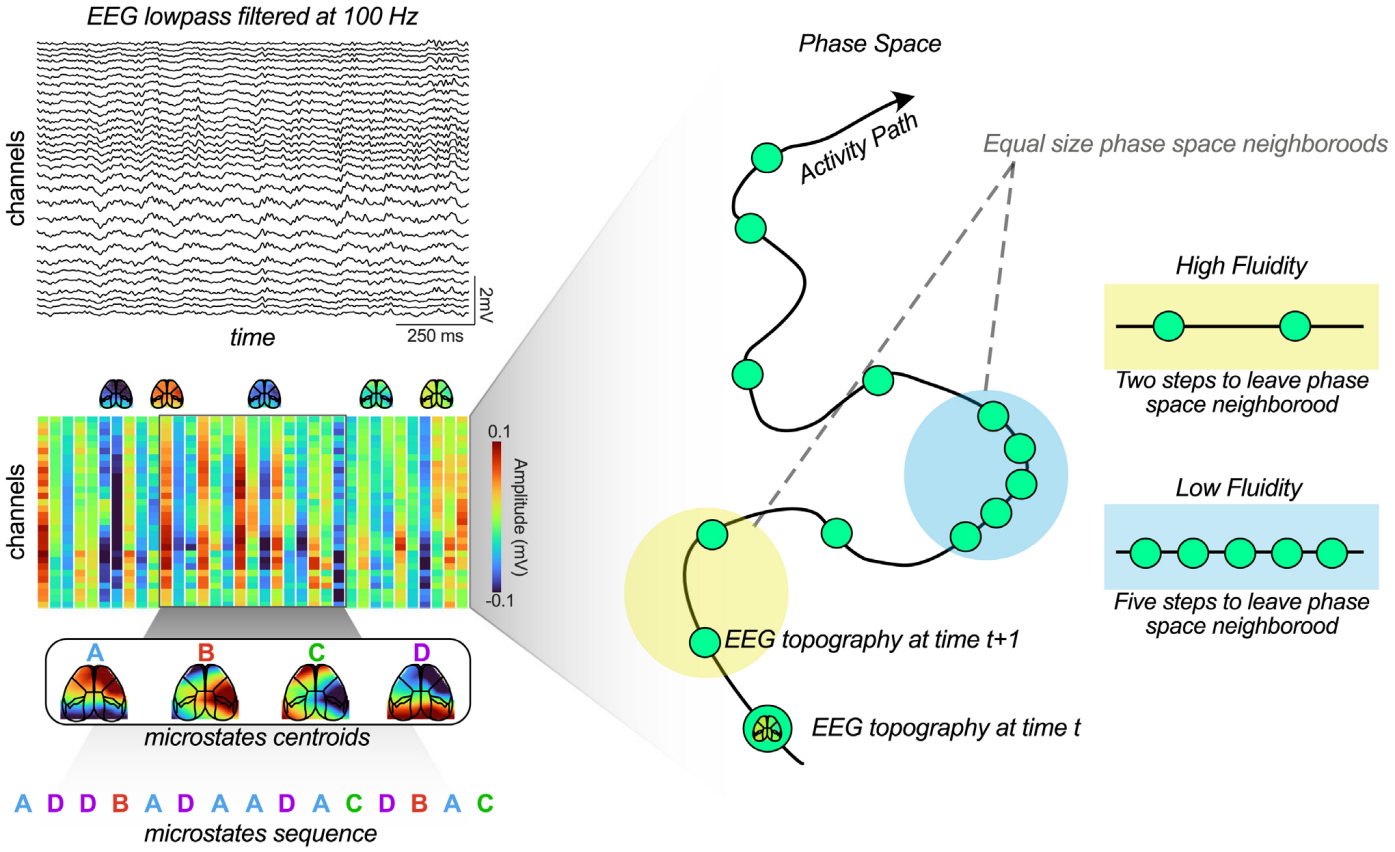
Analysis of global EEG brain dynamics. Top left, example of 30-channel hdEEG recorded during behavior. Middle, hdEEG is then coarse grained with 40 ms window generating 30-channel amplitude vectors over time. Right, Representation of EEG topography evolution in phase space, where each point corresponds to a 30-channel topography vector at a given time, as shown in the middle panel. Fluidity quantifies the temporal clustering of these topographies in phase space: when successive topographies are distant, fluidity is high (yellow); when successive topographies remain close, fluidity is low (blue). Bottom left, k-means clustering of 30 channels topography vectors identifies microstate centroids and sequences.

Microstates fluidity and complexity were analyzed using four-way ANOVA (factor: genotype, phase, clusters, task; see Supplementary Appendix Fig. A2). Post hoc tests were used when necessary, using a Bonferroni correction for multiple comparison. As the factor phase (sampling/test) & clusters showed no significant interaction with other factors, to reduce dimensions, analyses were further conducted on concatenated EEG between sampling and test phase, and microstates fluidity and complexity averaged over the number of microstates extracted with a two-way ANOVA (factor: genotype, task; Fig. 2). Beside similar two-way ANOVA (Fig. 5), post-vGENUS results were also analyzed with a repeated four-way ANOVA (factor: genotype, task, clusters, vGENUS, see Supplementary Appendix Fig. A5).

#### 2.6.4 Dynamics fluidity

EEG fluidity was computed on EEG time series concatenated between sampling and test. Average distributions were then compared two by two using bootstrapped Kolmogorov–Smirnoff (KS) statistics to probe whether the two distributions had significantly shifted mean and range, respectively, to a null hypothesis of no shift.

Using a Montecarlo procedure (pre-implemented in Matlab via the randsample function with a controlled random number stream), we redraw 5000 random samples according to each of the distributions of fluidity to compare (modeled as histograms with a resolution of 200 uniform-width bins, with different histograms for different genotypes and conditions before or after vGENUS). We performed the random subsampling of these large Montecarlo samples, generating reduced bootstrap with replacements replicas each including 500 resampled observations, under two alternative hypotheses. In a H1 hypothesis of association between fluidity and genotype and condition, subsampling was performed separately over each of the Montecarlo sample specific to the different cases. In a H0 hypothesis of lack of association, we merged the Montecarlo samples for the two genotypes and generated two random subsamples from this common merged sample. We then computed KS statistics between the two subsamples, quantifying over bootstrap replicas the distribution of KS statistics between fluidity distributions for different genotypes, under both the H1 and H0 hypotheses. In the Results section, we communicate the mean and standard deviation of KS statistics across iterations in the association hypothesis. Statistical significance of the difference between genotypes was assessed by comparing the H1 distribution of KS divergence values with the chance-level distribution under the H0 hypothesis, estimating a p-value based on the fraction of overlap between the two distributions, and corrected for multiple comparison with a Bonferroni correction. Note that this procedure is more conservative than the classical testing based on KS statistics non-parametric comparison.

#### 2.6.5 Continuous dynamics-microstates fluidity correlation

For each animal OiP and NOR preGENUS, mean dynamics fluidity was computed as the average of the EEG dynamics fluidity previously computed, mean microstates fluidity and complexity were computed by averaging microstates fluidity or complexity obtained with three to eight clusters to obtain a single value. Pearson correlations were then computed between mean dynamics fluidity and mean microstates fluidity or mean dynamics fluidity and mean microstates complexity taking all animals during OiP and NOR.

#### 2.6.6 Microstates sequence entropy for fluidity decile

To assess the potential link between specific microstate labels and EEG dynamics fluidity, the entropy of microstate sequences was computed across deciles of fluidity. For each animal, the dynamics fluidity time series was divided into 10 deciles. Microstate sequences were then extracted separately for each decile taking the four-cluster segmentation. The entropy of each microstate sequence was computed using the following formula:

$$H\left(fluidity\;decile\right)=\;-\;\sum_{k=1}^{4}p_{k}log_{2}\left(p_{k}\right),$$

where p(k) denotes the probability of occurrence of microstate k within the given decile.

## 3 Results

### 3.1 Young dKI mice display memory deficits in complex tasks before amyloid plaque onset

We first evaluate the extent of amyloid pathology in 4-month-old dKI mice. To this aim, we performed 6E10 immunohistochemistry on brain sections from 4 WT and 4 dKI mice (Fig. 1A, B; 2 males and 2 females per group). Across 262 brain slices from dKI mice, we detected only one plaque, in stark contrast to 12-month-old dKI positive controls (1 male, 1 female), where we observed a ratio of more than 10 plaques per slice. These results confirm that 4-month-old dKI mice do not yet exhibit significant amyloid plaque deposition.

We next assessed the memory performance of dKI mice at this pre-plaque stage. The Novel Object Recognition (NOR) task with a 24-hour delay, a standard test for evaluating recognition memory (Grayson et al., 2015), revealed no significant deficits in young dKI mice (Fig. 1C; Supplementary Appendix Fig. A1). This indicates that long-term recognition memory remains intact at this stage, unlike in mouse models with established amyloid pathology (Taglialatela et al., 2009). However, when we employed a more complex memory task taxing short-term associative memory, the Object in Place (OiP) task, we detected subtle but significant early memory deficits in dKI mice. Importantly, these impairments occurred independently of any alterations in locomotor speed or head movements (Fig. 1D; see Supplementary Appendix Fig. A1).

These findings, consistent with our previous work (Borcuk et al., 2022), demonstrate that 4-month-old dKI mice exhibit measurable memory impairments in complex tasks before the development of significant amyloid plaque pathology. This provides a critical window to study early brain network alterations associated with AD and to test potential early interventions before substantial pathology develops.

### 3.2 Global brain dynamics of dKI mice are altered before amyloid plaque onset and spectral slowing

To investigate whether these early memory deficits were associated with alterations in global brain dynamics, we first focused our analysis on the OiP task, where we observed cognitive impairments. We recorded high-density EEG during the performance of the task and characterized global brain dynamics using an unbiased approach, the EEG dynamics fluidity. This method treated multivariate EEG time series as trajectories in a high-dimensional space of brain activity topographies (30 dimensions, corresponding to the number of hdEEG channels). For each point, we computed dynamics fluidity (Faranda et al., 2017), a metric related to the time the system takes to leave a neighborhood of the visited point in the space of dynamic configurations (Fig. 2, right; Supplementary Appendix Fig. A2). This quantity, grounded in concepts from the statistical theory of extreme events (Lucarini et al., 2012) and previously used in the analysis of climatic time series (Faranda et al., 2017), offers important advantages on more classic metrics (Grassberger & Procaccia, 1983) of dynamic stability as it can be properly estimated from a considerably lower amount of data.

**Fig. 3.**
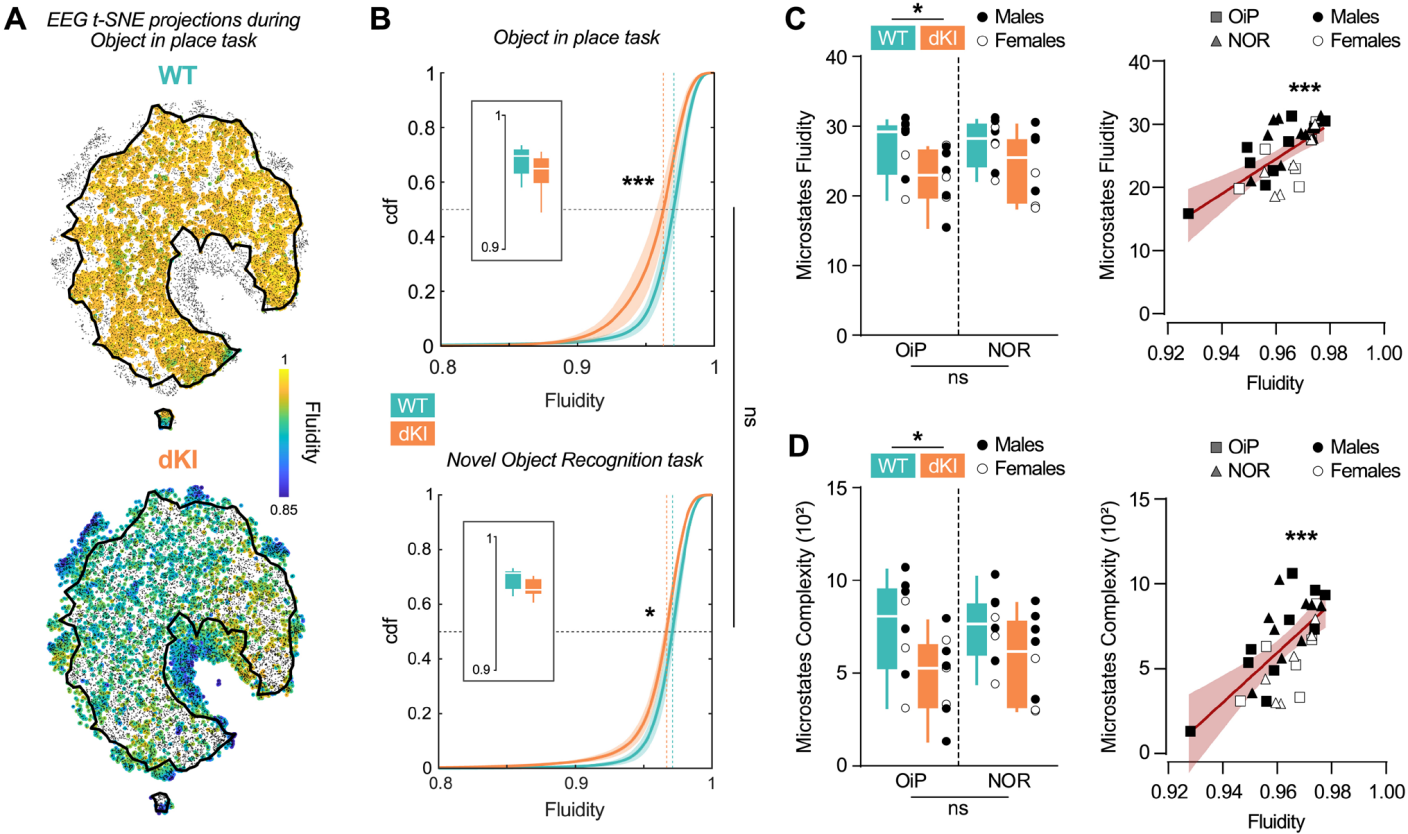
Global brain dynamics of dKI mice is altered before amyloid plaques onset. (A) t-SNE projection of 30 channels EEG topography vectors from a representative WT and dKI mouse during OiP task, color coded by EEG dynamics fluidity. Gray dots represent the full distribution of both genotypes, black line represents the borders of landscape occupied by WT points. (B) A shift between the cumulative distribution functions (cdf) of the EEG dynamics fluidity distributions for WT (blue) and dKI (orange) mice during OiP (top) and NOR (bottom) task (n = 8 per group) indicated reduced dynamics fluidity in dKI mice in OiP (KS = 0.2283 ± 0.0277, p < 0.001, here and for all other KS statistics in the following measured from a bootstrap comparison between association and null hypothesis, see Methods), and NOR (KS = 0.1626 ± 0.0279, p = 0.026). Data are presented as mean ± s.e.m over animals. Dotted lines show distribution medians. Box displays individuals mean dynamics fluidity distributions. (C) Microstates fluidity during OiP and NOR task for WT and dKI mice (left, n = 8 per group). Two-way ANOVA indicate only a genotype effect (F(1, 28) = 6.656, p = 0.015), indicating a reduced microstates fluidity in dKI mice for the two tasks. Box ranges from 25 to 75 percentile and whiskers for minimum to maximum values, median is represented by white line, individual points are displayed in black for males and white for females. Microstates fluidity correlates with mean dynamics fluidity over the two different tasks (right, R² = 0.4539, p < 0.001) and per task (Results not shown: OiP, squares, R² = 0.5665, p < 0.001; NOR, triangles, R² = 0.3141, p = 0.0239), individual points are displayed in black for males and white for females. (D) Microstates sequence complexity during OiP and NOR task for WT and dKI mice (left, n = 8 per group). Two-way ANOVA indicate only a genotype effect (F(1, 28) = 7.522, p = 0.011) indicating a reduced microstates sequence complexity in dKI mice for the two tasks. Box ranges from 25 to 75 percentile and whiskers for minimum to maximum values, median is represented by white line, individual points are displayed in black for males and white for females. Microstates fluidity correlates with mean dynamics fluidity over the two different tasks (right, R² = 0.4371, p < 0.001) and per task (Results not shown: OiP, squares, R² = 0.5248, p = 0.0015; NOR, triangles, R² = 0.3015, p = 0.0276), individual points are displayed in black for males and white for females.

Using t-SNE (Maaten & Hinton, 2008), a nonlinear, distance-preserving embedding method, we visualized these brain activity configurations as points in a two-dimensional space with their respective fluidity value (Fig. 3A). While the overall shape of the sampled manifold was similar across genotypes, the average distribution of dynamics fluidity across the manifold was significantly lower in dKI mice (Fig. 3B, top; KS = 0.2283 ± 0.0277, p < 0.001). This reduction was associated with dKI mice exploring dynamic configurations not observed in WT mice, potentially corresponding to states with abnormally low fluidity (Fig. 3A, bottom; where the border of WT landscape appears as a black line on the dKI landscape). These findings suggest that by 4 months of age, dKI mice experience a global reduction of fluidity and events where brain dynamics transiently exhibit highly reduced fluidity.

To determine whether these alterations in brain dynamics were specific to tasks showing cognitive deficits, we also analyzed dynamics fluidity during the NOR task performance. Notably, dynamics fluidity was also significantly lower in dKI (Fig. 3B, bottom; KS = 0.1626 ± 0.0279, p = 0.026), despite the absence of observable memory deficits in this task. We found no significant differences in dynamics fluidity between the two tasks for either WT (KS = 0.0558 ± 0.026, p = 1) or dKI mice (KS = 0.1296 ± 0.0263, p = 0.1468). This suggests that reduced dynamics fluidity is a general feature of dKI mice, independent of task-specific demands, and may serve as a sensitive early indicator of AD-related brain changes. To further support this idea, we investigated the time-resolved relationship between EEG dynamics fluidity and fine-grained behavioral and locomotor variables. First, we found that EEG dynamics fluidity did not exhibit any spatial pattern related to the animal’s position in the Open Field (Supplementary Appendix Fig. A3A). Moreover, mutual information analyses revealed only a weak association between EEG dynamics fluidity and either locomotor speed or head movements (Supplementary Appendix Fig. A3B, C).

To corroborate these findings, we conducted EEG microstate analyses (Michel & Koenig, 2018). Microstates represent a small number of stereotypical hdEEG topographies, extracted via unsupervised clustering. Continuous recordings were then converted into sequences of symbolic labels indicating the microstate closest to the current topography (Fig. 2, bottom). From these sequences, we calculated a fluidity-equivalent measure by quantifying the inverse of the probability to remain in the same microstate from time *t* to *t*+1. This measure was computed for several microstates numbers (from 3 to 8) and then averaged to generate a single value. Microstates fluidity was significantly reduced in dKI mice in both OiP and NOR tasks compared with WT (Fig. 3C; Two-way ANOVA, F(1, 28) = 6.656, p = 0.015) and showed a strong correlation with the previously computed dynamics fluidity (Fig. 3C; R² = 0.4539, p < 0.001).

In addition, we assessed the complexity of microstate sequences using a minimum description length approach, which has previously been shown to be affected in AD (Tait et al., 2020). Consistent with the microstates fluidity results, microstate sequence complexity was significantly reduced in dKI mice during both OiP and NOR tasks compared with WT mice (Fig. 3D; Two-way ANOVA, F(1, 28) = 7.522, p = 0.011) and also correlated strongly with dynamics fluidity (Fig. 3D; R² = 0.4371, p < 0.001). These results were robust, remaining independent of the average number of microstates extracted (see Supplementary Appendix Fig. A4A, B), microstates self-repetitions (see Supplementary Appendix Fig. A4C), and were confirmed using an alternative EEG microstates extraction method (see Supplementary Appendix Fig. A5). We also remark that fluidity drops where not restrained to the occurrence of specific microstates but occur with nearly equal likelihood across any of the extracted microstates. This can be quantified by computing the entropy of the probability of visiting different microstates as the function of the fluidity quantile (see *Materials and Methods*). For all quantiles, and notably for the lowest quantile associated with extremely low fluidity values, the microstate entropy always remained at less than 10% away the theoretically maximum value, corresponding to perfectly uniform probability of visiting any microstate (Supplementary Appendix Fig. A6).

**Fig. 4.**
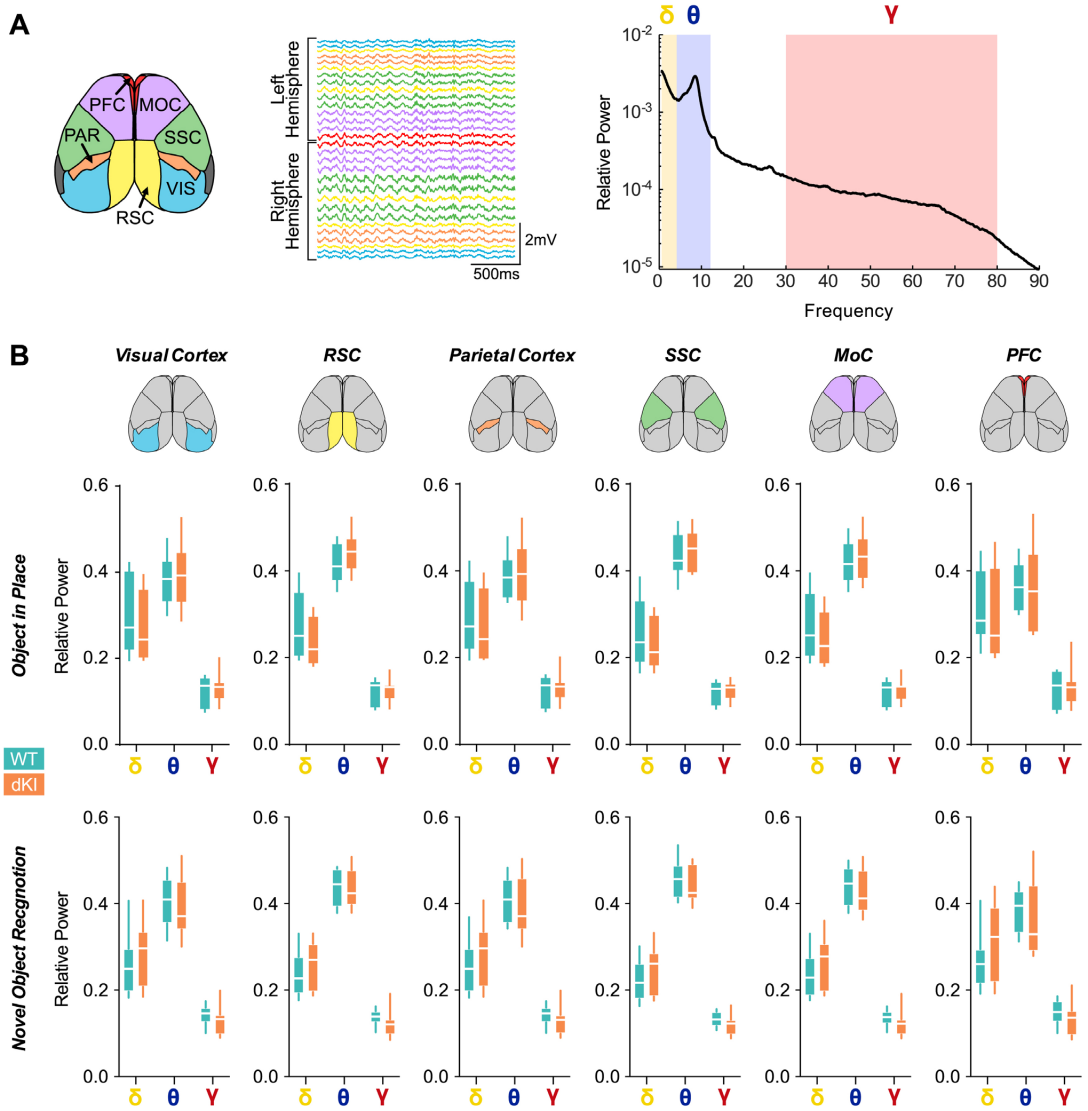
dKI mice show no spectral alterations in both tasks. (A) Cortical parcelation map (*left*) of the 30 channels hdEEG based on Allen Atlas, allowing the nomenclature of the electrodes and parcelation of the data into these following the cortical territory over which the electrode is located: visual cortex (VIS, blue); retrosplenial cortex (RSC, yellow); parietal cortex (PAR, orange); somato sensory cortex (SSC, green); motor cortex (MOC, purple); prefrontal cortex (PFC, red). The power of these channels is averaged over regions for next analyses. Relative power spectrum (*right*) is computed for each cortical territory and the relative power of delta (0.5–4 Hz, yellow), theta (4–12 Hz, blue), and gamma (30–80 Hz, red) is assessed. (B) Relative power for delta (yellow), theta (blue), and gamma (red) bands during the performance of OiP (*Top*) and NOR (*bottom*) tasks over different cortical territories. Multiple t-test between genotypes for each region and frequency bands show no difference of spectral relative power between genotypes.

Furthermore, to assess whether the reduction in brain dynamics fluidity observed in dKI mice could be linked to spectral alterations, we analyzed the relative power of delta (0.5–4 Hz), theta (4–12 Hz), and gamma (30–80 Hz) frequency bands. These analyses were conducted across cortical regions defined by electrode placement based on the Allen brain parcellation (Fig. 4A), during both the OiP and NOR tasks. As expected, both WT and dKI mice exhibited a dominance of theta-band activity, consistent with the known role of theta rhythms in rodents during navigation, active wakefulness, and memory task performance (Fig. 4B). Strikingly, we found no significant alterations of average relative power in any frequency bands nor brain regions in dKI mice during task performance (Fig. 4B). While subtler spectral changes cannot be entirely ruled out, these results suggest two possible interpretations: either reduction in dynamics fluidity emerges before the onset of spectral alterations, or the global, system-level approach of dynamics fluidity provides greater sensitivity than region-specific and frequency band-specific analyses in detecting early dysfunction in brain network activity. Collectively, these findings support the concept that alterations in brain dynamics, as measured by dynamics fluidity, represent an early marker of AD pathology that precedes both amyloid plaque formation and the classically reported EEG abnormalities such as spectral slowing.

### 3.3 Global brain dynamics of dKI mice are unaltered during sleep

**Fig. 5.**
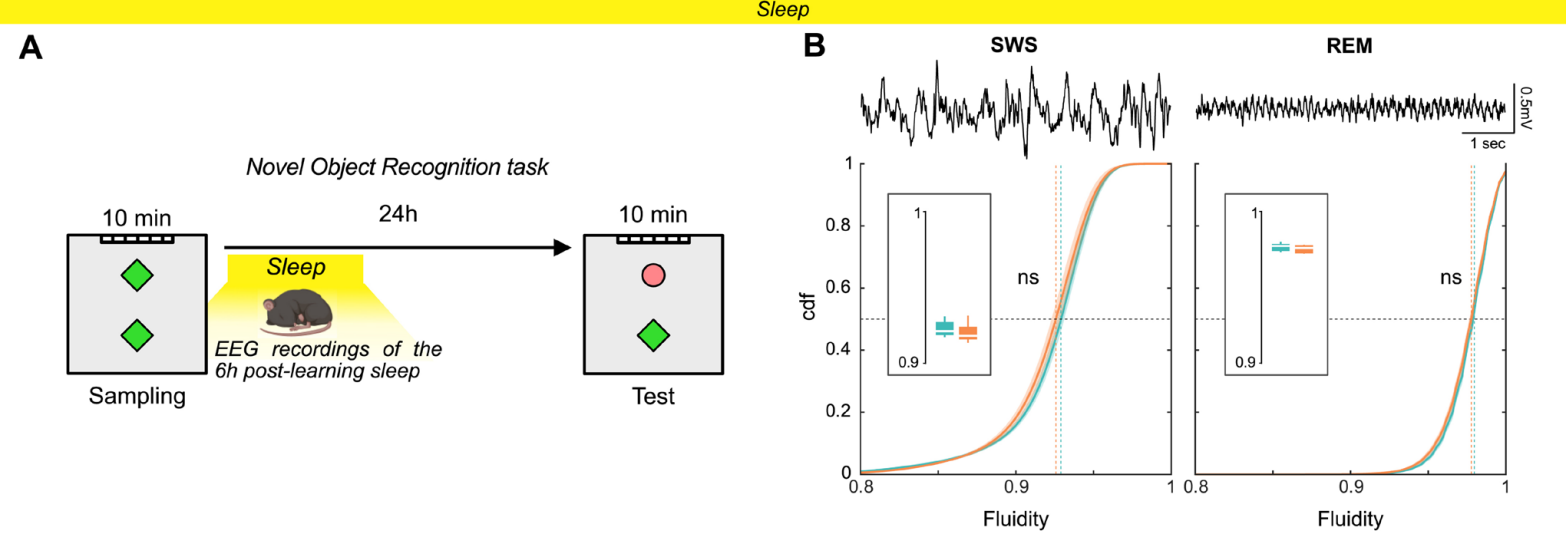
Global brain dynamics of dKI mice is unaltered during sleep. (A) EEG was recorded during the first 6 hours post-learning of the NOR task. (B) Top, example EEG signals during slow-wave sleep (SWS, left) and REM sleep (right). Bottom, no significant differences in EEG dynamics fluidity between WT and dKI mice during SWS or REM. Data are presented as mean ± s.e.m over animals. Dotted lines show distribution medians. Box displays individuals mean dynamics fluidity distributions.

Given that global brain dynamics were altered in both OiP and NOR tasks, yet dKI mice showed memory deficits only in the OiP task, we sought to understand this discrepancy. We hypothesized that the difference might be attributed to either the complexity of the task (NOR being simpler than OiP) or the difference in delay between tasks. The 24-hour delay in the NOR task allows for sleep during the intertrial interval, potentially enabling offline memory consolidation. To address whether brain dynamics are altered during sleep, we analyzed dynamics fluidity during rapid eyes movements (REM) and slow-wave sleep (SWS) in the hours following learning in the 24-hour NOR task (Fig. 5A), as these states are critical for memory consolidation (Boyce et al., 2016; Lu et al., 2018).

Intriguingly, we found that both SWS (KS = 0.0876 ± 0.0273, p = 0.3122) and REM (KS = 0.0653 ± 0.0232, p = 0.617) exhibited similar dynamics fluidity between dKI and WT mice (Fig. 5B). Furthermore, spectral analysis revealed no major alterations in the sleep EEG profiles of dKI mice (Supplementary Appendix Fig. A7). This preserved dynamics fluidity during sleep, in contrast to the altered dynamics during wakefulness, may explain the differential impact on behavior observed between the two memory tasks. Specifically, while the OiP task relies on online associative processes that may be disrupted by transient reductions in dynamics fluidity, the 24-hour NOR task may benefit from offline memory consolidation during sleep.

The intact dynamics during sleep might, therefore, help compensate for errors introduced by altered dynamics during wakefulness.

### 3.4 vGENUS differentially entrains cortical regions at 40 Hz and increases brain dynamics in dKI mice

**Fig. 6.**
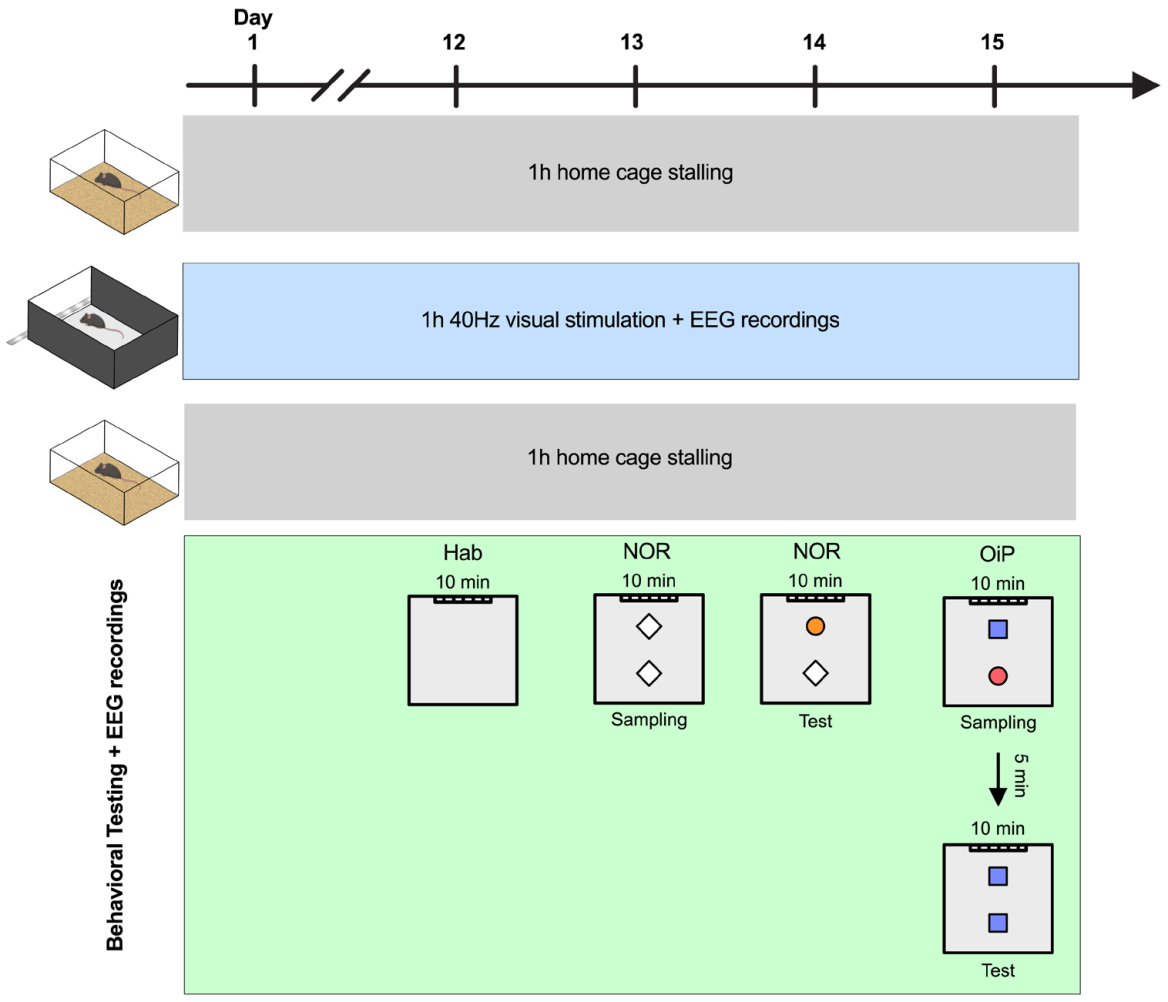
Visual GENUS stimulation protocol. Every day for 15 days, mice are placed in the stimulation room in their home cage for a 1-h stalling. They are then placed in the stimulation cage, which have three of its four walls painted black to restrict light exposure to the side facing the stimulation setup. The cage contains no bedding, food, or water. Mice undergo 1 hour of light stimulation while EEG is recorded. After stimulation, they are returned to their home cage for another 1-hour stalling period. Starting on day 12, behavioral tasks were introduced following the post-stimulation stalling period: open-field habituation on day 12, the Novel Object Recognition (NOR) task on days 13 and 14, and the Object-in-Place (OiP) task on day 15.

Given the reduced dynamics fluidity observed in dKI mice, we next investigated whether vGENUS could potentially modulate these altered brain dynamics. We conducted 2 weeks of daily 1-hour vGENUS sessions (Fig. 6) and analyzed hdEEG for 10 minutes during stimulation on both the first (Day 1) and last (Day 15) day of the protocol to assess the immediate and long-term effects of the intervention.

**Fig. 7.**
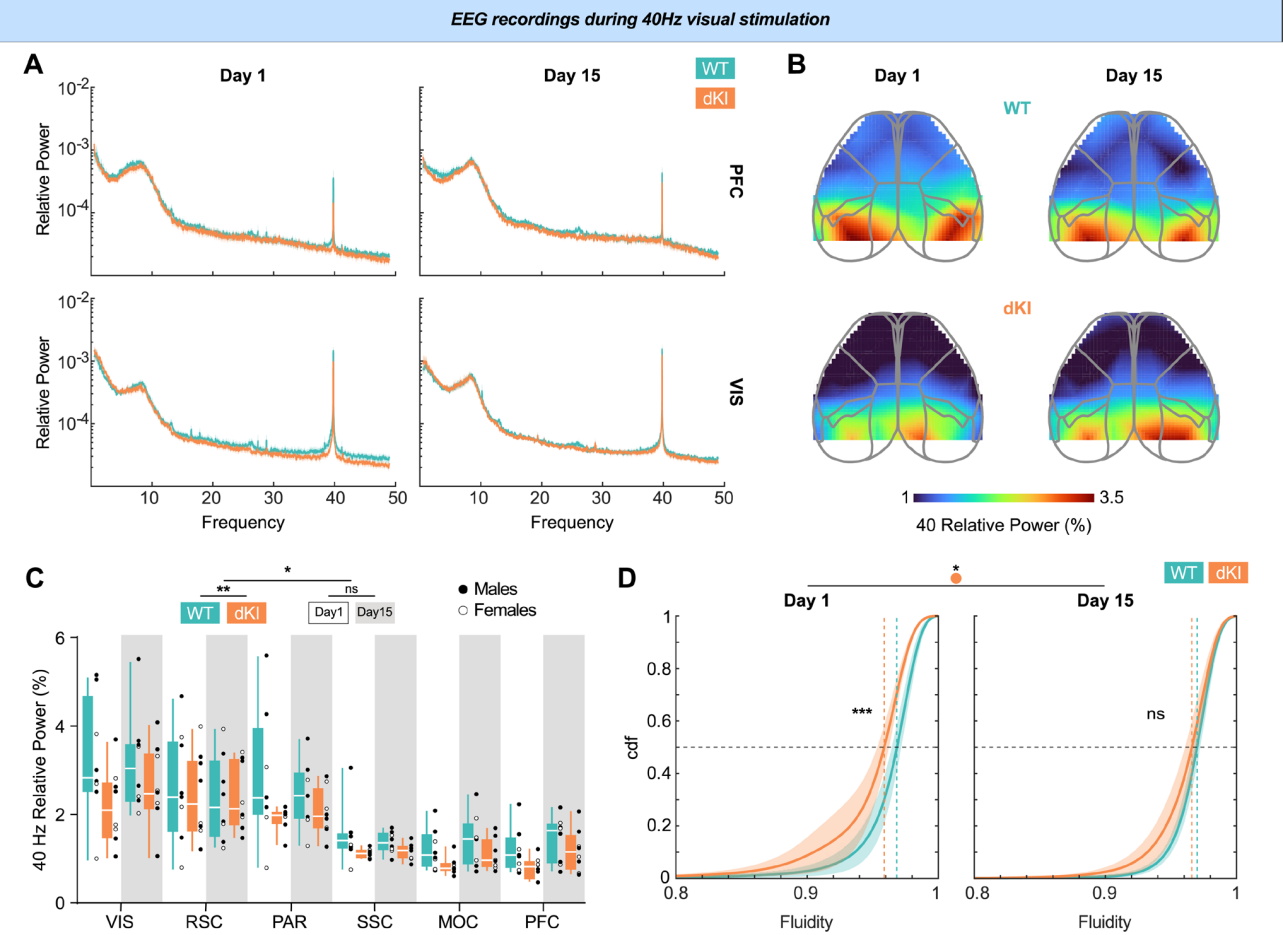
vGENUS differentially entrain cortical territories at 40 Hz and increase brain dynamic in dKI mice. (A) Relative power spectrum of PFC *(Top)* and VIS *(bottom)* during first *(left)* and last *(right)* day of 40 Hz vGENUS stimulation for one WT (blue) and one dKI (orange) mouse. A peak at 40 Hz can be observed in both mice, both regions and both first and final day meaning a cortical entrainment by the vGENUS stimulation. (B) Cortical map of 40 Hz proportion of the power spectrum (40 Hz power/all power spectrum) during first (left) and last (right) day of stimulation for one WT (top) and one dKI (bottom) mouse. As expected, visual areas are the more entrained by 40 Hz, this entrainment appears lower in the dKI mouse. (C) To quantify observations of previous maps, we measured the relative 40 Hz power across different cortical territories during the first (no background) and last (gray background) day of stimulation for WT (n = 8, blue) and dKI (n = 8, orange) mice. Two-way ANOVA on repeated measures revealed a significant interaction between the day of stimulation and genotype (F(1, 84) = 4.619, p = 0.034). Post hoc tests showed that the 40 Hz proportion was lower in dKI mice on Day 1 (t(84) = 3.265, p = 0.009), and no difference between genotypes on Day 15 (t(84) = 1.975, p = 0.309). Box ranges from 25 to 75 percentile and whiskers for minimum to maximum values, median is represented by white line, individual points are displayed in black for males and white for females. (D) EEG fluidity distribution during first (left) and last (right) day of vGENUS stimulation for WT (blue, n = 8) and dKI (orange, n = 8) mice showed a lower EEG fluidity at Day 1 for dKI mice (KS = 0.2548 ± 0.0284; p < 0.001) which normalized to WT levels at Day 15 (KS = 0.1459 ± 0.0266; p = 0.066) after a specific increased fluidity in dKI mice (KS = 0.1587 ± 0.0275; p = 0.03). Data are presented as mean ± s.e.m over animals. Dotted lines show distribution medians.

First, we examined the effects of vGENUS on cortical activity. A 40 Hz peak in the power spectrum was observed across regions for both genotypes, with a higher relative 40 Hz power in occipital (i.e., visual) regions (Fig. 7A, B). However, the relative 40 Hz power during stimulation differed between genotypes and between Day 1 and Day 15 of the stimulation protocol (Fig. 7C; three-way repeated ANOVA, F(1, 84) = 4.619, p = 0.034). Specifically, relative 40 Hz power during stimulation was lower in dKI mice at Day 1 (t(84) = 3.265, p = 0.009) but not significantly different from WT levels at Day 15 (t(84) = 1.975, p = 0.309). These findings suggest that the cortical response to 40 Hz visual stimulation is initially reduced in young dKI mice but changes after the 15-day vGENUS protocol.

Given the ongoing debate on the significance of 40 Hz stimulation, we next assessed whether vGENUS impacted global brain dynamics beyond its effects on 40 Hz power. To this end, we computed dynamics fluidity during the stimulation periods on both Day 1 and Day 15. On Day 1, dKI mice exhibited significantly lower dynamics fluidity than WT mice (Fig. 7D; KS = 0.2548 ± 0.0284, p < 0.001). However, after 15 days of stimulation, dynamics fluidity in dKI mice increased (KS = 0.1587 ± 0.0275, p = 0.03), reaching levels comparable with WT mice (KS = 0.1459 ± 0.0266, p = 0.066).

These results reveal a dissociation between acute and chronic effects of vGENUS. While a single stimulation session (Day 1) successfully entrained 40 Hz power across multiple electrode sites, this acute entrainment did not immediately restore the impaired global brain dynamics in dKI mice. In contrast, after 15 days of repeated stimulation, dynamics fluidity in dKI mice significantly increased to levels comparable with WT mice. These findings highlight the importance of chronic stimulation protocols and indicate that the therapeutic mechanisms of vGENUS likely extend beyond simple entrainment of cortical oscillations to involve broader changes in network dynamics, reprogrammed beyond the immediately induced stimulation effects.

### 3.5 Increased brain dynamics in dKI mice persist after end of stimulation and restore memory performance

**Fig. 8.**
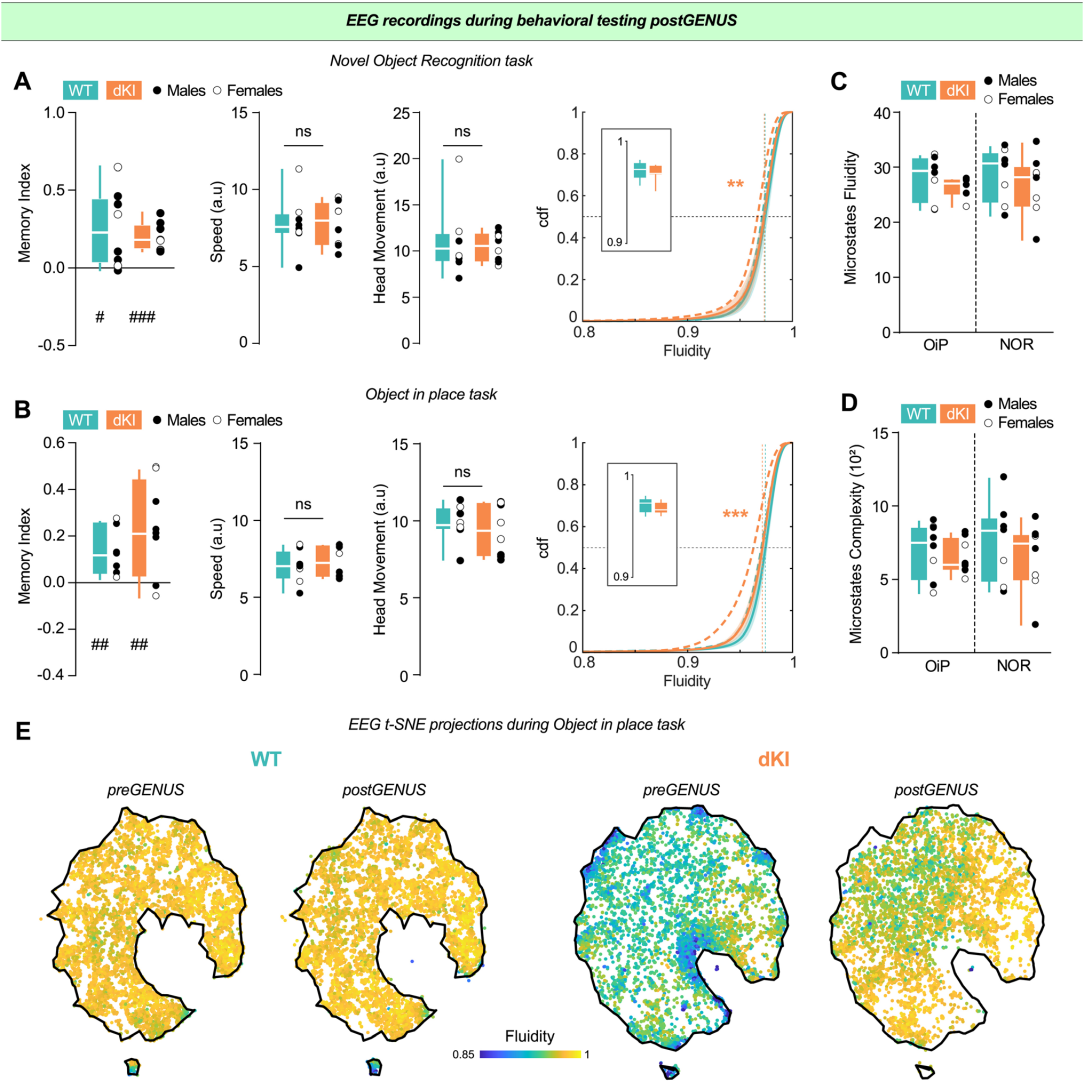
Increased brain dynamics in dKI mice remain after end of stimulation and restore memory performances in dKI mice. (A) Post-GENUS NOR memory index (left), speed (center left), and head movement (center right) for WT (n = 8, blue) and dKI (n = 8, orange) mice. Both WT and dKI show memory performances higher than chance levels (one-sided t-test against chance, #: p < 0.05, ##: p < 0.01, ###: p < 0.001), while showing no differences in speed and head movement between genotypes. Box ranges from 25 to 75 percentile and whiskers for minimum to maximum values, median is represented by white line, individual points are displayed in black for males and white for females. Brain dynamics fluidity shows no more genotype differences (right, KS = 0.0798 ± 0.0235, p = 0.6405) due to a significant increase in dynamics fluidity in dKI mice after vGENUS (KS = 0.2137 ± 0.0293, p = 0.0042). Data are presented as mean ± s.e.m over animals. Dotted lines show distribution medians. Box displays individuals mean dynamics fluidity distributions. (B) Post-GENUS OiP memory index (left), speed (center left) and head movement (center right) for WT (n = 8, blue) and dKI (n = 8, orange) mice. Both WT and dKI show memory performances higher than chance levels (one-sided t-test against chance, #: p < 0.05, ##: p < 0.01, ###: p < 0.001), while showing no differences in speed and head movement between genotypes. Box ranges from 25 to 75 percentile and whiskers for minimum to maximum values, median is represented by white line, individual points are displayed in black for males and white for females. Brain dynamics fluidity show no more genotype differences (right, KS = 0.1298 ± 0.0270, p = 0.0999) due to a specific increase in dKI mice (KS = 0.237 ± 0.0283, p < 0.001) after vGENUS. Data are presented as mean ± s.e.m over animals. Dotted lines show distribution medians. Box displays individuals mean dynamics fluidity distributions. (C) Microstates fluidity during OiP and NOR task for WT and dKI mice post-vGENUS (n = 8 per group). Box ranges from 25 to 75 percentile and whiskers for minimum to maximum values, median is represented by white line, individual points are displayed in black for males and white for females. (D) Microstates sequence complexity during OiP and NOR task for WT and dKI mice post-vGENUS (n = 8 per group). Box ranges from 25 to 75 percentile and whiskers for minimum to maximum values, median is represented by white line, individual points are displayed in black for males and white for females. (E) t-SNE projection of hdEEG multidimensional vectors from a representative WT (left) and dKI (right) mouse during OiP task before and after vGENUS, color-coded by EEG dynamics fluidity. Black line represents the border of the activity landscape before vGENUS for each genotype.

Since memory deficits in young dKI mice were likely linked to altered brain dynamics, we examined whether vGENUS-mediated restoration of brain dynamics would also improve memory performance. At the end of the 2 weeks of vGENUS, NOR and OiP tasks were performed in the same order as before vGENUS (Fig. 6). vGENUS did not affect performance, speed, or head movements in the NOR task, as dKI mice showed no impairments both before and after vGENUS (Fig. 8A; see Supplementary Appendix Fig. A8E–H). However, there was no longer a significant difference in EEG dynamics fluidity between genotypes (Fig. 8A, right; KS = 0.0798 ± 0.0235, p = 0.6405) due to a significant increase in EEG dynamics fluidity in dKI mice after vGENUS (KS = 0.2137 ± 0.0293, p = 0.0042).

Thus, the vGENUS-induced increase in brain dynamics appears to persist even 1 hour after stimulation sessions, suggesting a stable shift toward more physiological brain dynamics that could benefit cognitive performance. Indeed, after 2 weeks of vGENUS, dKI mice no longer showed deficits in the OiP task (Fig. 8B, left; see Supplementary Appendix Fig. A8A–D). Similarly to the NOR task, EEG dynamics fluidity during the OiP task showed no significant difference between genotypes after vGENUS (Fig. 8B, right; KS = 0.1298 ± 0.0270, p = 0.0999) due to a specific increase in dKI mice (KS = 0.237 ± 0.0283, p < 0.001).

Discretized microstates analysis supported these findings, showing no significant difference between genotypes in either microstates fluidity (Fig. 8C; see Supplementary Appendix Fig. A9A) or microstates sequence complexity (Fig. 8D; see Supplementary Appendix Fig. A9B) after 2 weeks of vGENUS.

Our results demonstrate that vGENUS restores memory performance in the OiP task while simultaneously increasing dynamics fluidity in dKI mice, with effects persisting beyond the immediate stimulation period. Notably, this increased fluidity represents a general shift toward a more dynamic state rather than being confined to specific low-fluidity moments, as evidenced by an upward shift in the entire distribution of fluidity values (see Supplementary Appendix Fig. A10). This shift appears to reflect a fundamental change in basal brain state rather than task-specific alterations, as we observed no strong relationship between dynamics fluidity and behavioral variables (see Supplementary Appendix Fig. A11), implying that fluidity changes reflect thus genuine endogenous changes in brain dynamics, rather than indirect exogenous correlations. These findings support the concept of a global “thawing” of brain dynamics to a more fluid state resembling that of WT mice. In addition, this global fluidity increase appears to eliminate pathological configurations characterized by extremely low fluidity (Fig. 8E; where extreme low fluidity points present in pre-GENUS dKI mice are absent from the post-GENUS landscape; see also Supplementary Appendix Fig. A12).

Crucially, this enhancement of brain dynamics occurred independently of spectral changes, as we observed no effects on delta, theta, or, most notably, gamma oscillations during either the OiP or NOR task between pre- and post-vGENUS conditions (see Supplementary Appendix Figs. A13 and A14). This suggests that 40 Hz stimulation may exert its therapeutic effects through mechanisms beyond direct modulation of gamma oscillations.

Furthermore, vGENUS effects on dynamics fluidity appear specific to pathological states. Sleep dynamics, which were unaffected in dKI mice before treatment, showed no differences in fluidity after vGENUS (see Supplementary Appendix Fig. A15), although marginal vGENUS-induced changes in delta and theta power were observed in both genotypes (see Supplementary Appendix Figs. A16 and A17). This state-specificity suggests that vGENUS preferentially normalizes disrupted brain dynamics while having minimal impact on already-normal dynamical states, potentially indicating a ceiling effect on healthy global brain dynamics fluidity.

## 4 Discussion

Using high-density EEG recordings during specific memory tasks in a preclinical AD mouse model, along with the assessment of global brain dynamics through novel metrics originally developed in fields outside neuroscience (Faranda et al., 2017), we demonstrate that dKI mice exhibit early, awake state-specific alterations in brain dynamics associated with cognitive deficits in complex memory tasks. Importantly, these alterations occur before amyloid plaque onset, challenging the traditional amyloid-centric view of early AD pathology. However, we cannot rule out any implication of soluble Aβ, as slightly increased levels were already observed in 4-month-old dKI mice (Borcuk et al., 2022) and soluble Aβ has previously showed to induce hyperexcitability in early AD (Busche et al., 2012; Busche & Konnerth, 2016), which may possibly causally contribute to the alteration of cortical dynamics we are observing. Conversely, one could also hypothesize that amyloid aggregation and plaque formation further exacerbate alterations in brain dynamics fluidity. This idea is supported by prior findings showing that dKI mice exhibit more pronounced memory impairments at 6 months than at 4 months (Borcuk et al., 2022), and by our present results showing clear amyloid plaque deposition at 12 months. Nevertheless, our data demonstrate that alterations in EEG dynamics fluidity are already observable *in vivo* before the onset of amyloid pathology.

In addition, these changes in global brain dynamics fluidity precede significant shifts in the power of specific oscillatory frequency bands. EEG dynamics fluidity thus offers a more sensitive, unsupervised tool for detecting subtle global brain changes, making it particularly valuable for identifying early pathological alterations in clinical settings. Furthermore, we demonstrate that 2 weeks of daily vGENUS treatment not only restores these impaired dynamics to levels comparable with WT mice but also rescues memory deficits in the OiP task. Notably, these beneficial effects on brain dynamics persist beyond the immediate stimulation period and appear to be independent of spectral changes during task performance, suggesting that vGENUS may exert its therapeutic effects through broader mechanisms than direct modulation of oscillatory activity.

While global nonlinear brain dynamics have been previously studied using established metrics such as the Lyapunov exponent (Babloyantz & Destexhe, 1986; Stam, 2005; Wolf et al., 1985), these methods typically rely on long, stationary time series and an *a priori* understanding of the system’s governing equations, conditions that are not always met by experimental neuroscience data. In contrast, dynamics fluidity, as introduced here, is computed from shorter, empirical time series without requiring explicit modeling of the system. This makes it a more flexible approach, better suited to real-world data, particularly in translational and clinical contexts where access to long, stationary datasets is often limited.

Our results suggest that reduced brain dynamics fluidity in early-stage AD is indicative of a fundamental alteration in brain function. If we consider that cognitive performance relies on proper brain computation, this shift toward a less dynamic functional regime likely imposes constraints on the brain’s computational capacity. Building on Turing’s seminal intuition that computations are not performed by setting the system in a fixed state but rather by structurally transiting across sequences of changing states, we could even speculate that efficient brain computation similarly and inherently requires a flexible flow across system dynamics configurations. In this framework, reduced fluidity, as observed in dKI mice, may reflect diminished dynamic flexibility, ultimately limiting computational capacity. As a result, in tasks with low cognitive (and thus computational) demands, such as the NOR task, this reduced fluidity may still allow for adequate performance. However, when task complexity increases, as in the OiP task, which places higher demands on cognitive resources and online computations, these limitations become more apparent, leading to observable deficits.

These dynamical alterations could be present in several pathologies; however, dynamics fluidity shows promise as an early marker for AD. Given the non-invasive nature and accessibility of EEG, assessing brain dynamics fluidity may allow for earlier detection of AD, potentially identifying changes during the MCI and subjective cognitive decline (SCD) stages. During these early stages, patients report cognitive issues but show no deficits in standard neuropsychological tests, making the early detection of dynamic changes particularly valuable. We hypothesize that these patients may exhibit a reduced basal dynamics fluidity state compared with healthy aging, though their dynamics may still be sufficiently flexible to support performance on conventional memory assessments.

An easily implementable diagnostic tool for early-stage AD could significantly improve the timing and effectiveness of treatment interventions. The potential clinical translation of this approach warrants further investigation, including longitudinal studies in human populations to validate the predictive power of brain dynamics fluidity in AD progression. In addition, the potential inter-individual variability in brain dynamics fluidity may require standardization of evaluation methods in human studies, particularly in terms of electrode number and placement, as well as the cognitive tasks performed during recording.

Consistent with previous reports (Adaikkan et al., 2019; Martorell et al., 2019), we found that 2 weeks of vGENUS rescued AD-related memory deficits in dKI mice. However, as recent studies have highlighted (Soula et al., 2023; Yang & Lai, 2023), since 4-month-old dKI mice lack amyloid plaques, it is improbable that vGENUS targets amyloid pathology directly, or at least not exclusively or primarily. Instead, our data suggest that vGENUS restores healthy brain dynamics fluidity, an effect that persists even after stimulation ends. This finding, coupled to the absence of vGENUS effects on gamma spectral power during the task, points to a broader impact of vGENUS on brain dynamics beyond 40 Hz entrainment, which has been debated in recent literature (Schneider et al., 2023; Soula et al., 2023). Notably, the restoration of global brain dynamics was not immediate but required a chronic protocol, indicating that long-term processes rather than immediate brain entrainment likely drive these changes.

One potential explanation for the observed effects could be metabolic reorganization of brain networks. Indeed, vGENUS has been shown to entrain vascular reactions in the brain, notably increasing blood vessel diameter (Martorell et al., 2019; Murdock et al., 2024) and has already demonstrated beneficial effects in mouse models of stroke (Zheng et al., 2020). Given that cerebral hypoperfusion has been linked to AD development (Kalaria et al., 2012) and cerebrovascular dysfunctions are associated with cognitive impairments (Fouda et al., 2019), vGENUS-induced increases in brain blood flow could improve brain metabolism and, in turn, brain dynamics.

Another possibility is that vGENUS modulates specific neuromodulatory systems, especially since the reduction in brain dynamics fluidity observed in dKI mice is specific to the awake state. vGENUS showed no effect on brain dynamics during sleep, suggesting that the mechanism may involve specific wake-related neuromodulators such as the noradrenergic system, which is both an early target in AD pathology (Bekdash, 2021) and known to affect large-scale brain dynamics (Shine et al., 2021). Future research will be needed to clarify the precise mechanisms by which vGENUS exerts its effects. However, our results, along with previous findings, converge to suggest that vGENUS beneficial effects on global brain dynamics are not specific to AD, as it has also shown benefits in epileptic patients (Blanpain et al., 2024). This suggests that vGENUS may represent a promising non-invasive treatment option for a variety of neurological disorders by targeting and modulating large-scale brain dynamics. Specifically, it could trigger endogenous mechanisms for compensating early circuit dysfunction via a “reprogramming” of the working point of dynamic operation of brain networks (Petkoski et al., 2023; Stulz et al., 2024). In the future, a better mechanistic understanding of the effect of GENUS on dynamics will allow optimizing specifications of the treatment, such as sensory modality, protocol duration, and frequency of sensory stimulation. In this study, only 40 Hz visual stimulation was tested, based on prior work showing that beneficial effects in AD models were specifically associated with this frequency (Martorell et al., 2019). However, we cannot exclude the possibility that other stimulation frequencies may also modulate brain dynamics fluidity, even if they do not confer the same cognitive or clinical benefits. Future studies comparing multiple stimulation frequencies will be necessary to determine whether increased fluidity is a specific mechanism underlying the therapeutic efficacy of 40 Hz GENUS, or a more general response to rhythmic sensory stimulation.

While our study provides novel insights into early AD pathophysiology and potential interventions, it is important to keep in mind that the use of a mouse model, while allowing for precise control and manipulation, may not fully recapitulate human AD pathology. In addition, the behavioral task used provides a relatively coarse and generic index of memory performance, due to its acute nature. As a result, individual-level correlations between memory performance and EEG dynamics fluidity could not be meaningfully assessed. Future studies should address this by employing more specific and ecological behavioral paradigms that allow repeated trials and learning, such as a radial maze task (Douchamps et al., 2024), to better capture the relationship between memory and brain dynamics fluidity. Finally, other important aspects remain to be explored, as the eventual existence of sex-specific differences (here impossible to assess due to small sample size and limited statistical power) or the long-term effects of vGENUS beyond the 2-week period studied here.

In conclusion, our study, using a new unsupervised EEG analytical tool, reveals altered brain dynamics as an early marker of AD-related changes, detectable before significant amyloid plaque formation and correlating with subtle cognitive deficits. We demonstrate that vGENUS can restore these altered dynamics and ameliorate associated memory impairments. These findings not only provide new insights into early AD pathophysiology but also suggest novel approaches for early diagnosis and intervention in AD and potentially other neurological disorders characterized by disrupted brain dynamics.

## Supplementary Material

Supplementary Material

## Data Availability

The code used for preprocessing and analyses was implemented in MATLAB 2024a using the Chronux toolbox (http://chronux.org/) and custom scripts. Important custom-made code is available on the GitHub page of the laboratory https://github.com/FunSyCNRS. The raw data that support the findings of this study are available from the corresponding author upon reasonable request.
