# Supplementary material for: 40 Hz light stimulation restores early brain dynamics alterations and associative memory in Alzheimer’s disease model mice
Source: Imaging Neurosci (Camb). 2025 Jul 14;3:IMAG.a.70. doi: 10.1162/IMAG.a.70 (PMC12330864; doi:10.1162/IMAG.a.70)
Supplement: Supplementary Material [file IMAG.a.70_supp.pdf]

Supplementary Information for

40 Hz light stimulation restores early brain dynamics alterations and associative memory in Alzheimer's disease model mice

Matthieu Aguilera[1], Chantal Mathis[1], Karin Herbeaux[1], Amine Isik[1], Davide Faranda[2], Demian Battaglia[1,*] & Romain Goutagny[1,*]

[1] Université de Strasbourg, CNRS, Laboratoire de Neurosciences Cognitives et Adaptatives (LNCA), UMR 7364, F–67000 Strasbourg, France.

[2] Université Paris Saclay, CEA – CNRS – UVSQ, Laboratoire des Sciences du Climat et de l’Environnement (LSCE), F-91191 Gif-sur-Yvette, France

* Demian Battaglia, Romain Goutagny.

Email: dbattaglia@unistra.fr, goutagny@unistra.fr

This PDF file includes:

Appendix Figures A1 to A17

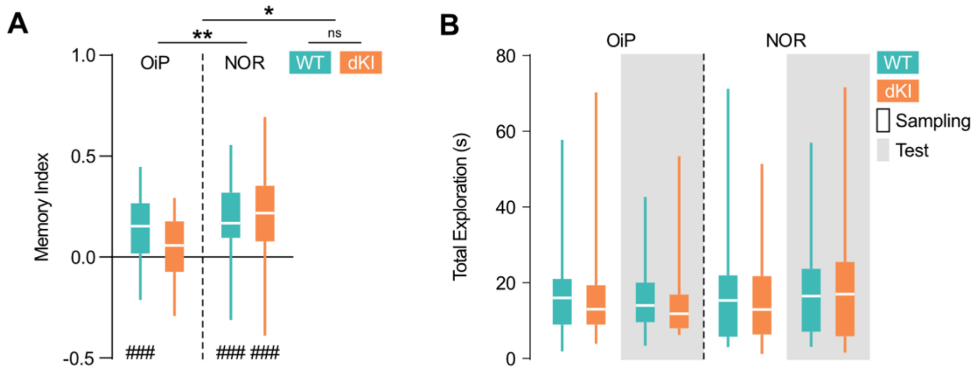

Appendix Fig. A1. dKI mice memory deficits in complex task are unrelated to exploration time. Box ranges from 25 to 75 percentile and whiskers for minimum to maximum values, median is represented by white line. (A) Memory Index for WT (blue, n = 35) and dKI (orange, n = 37) mices performing Object in Place (OiP) and Novel Object Recognition (NOR) Tasks. Two way ANOVA (factor: Task, Genotype) reveal a significant interaction between Genotype and Task ($F(1,140) = 5.17$, $p = 0.024$) and only dKI mice performing OiP showed performances not higher than chance level (one sided t-test against chance, # : $p < 0.05$, ### : $p < 0.001$). (B) Total exploration time of the two objects for WT and dKI mice during Sampling (no background) and Test (grey background) of OiP and NOR task. Three way ANOVA (factor: Genotype, Task, Phase) shows no significant effects, thus indicating no difference in exploration between genotypes in both tasks.

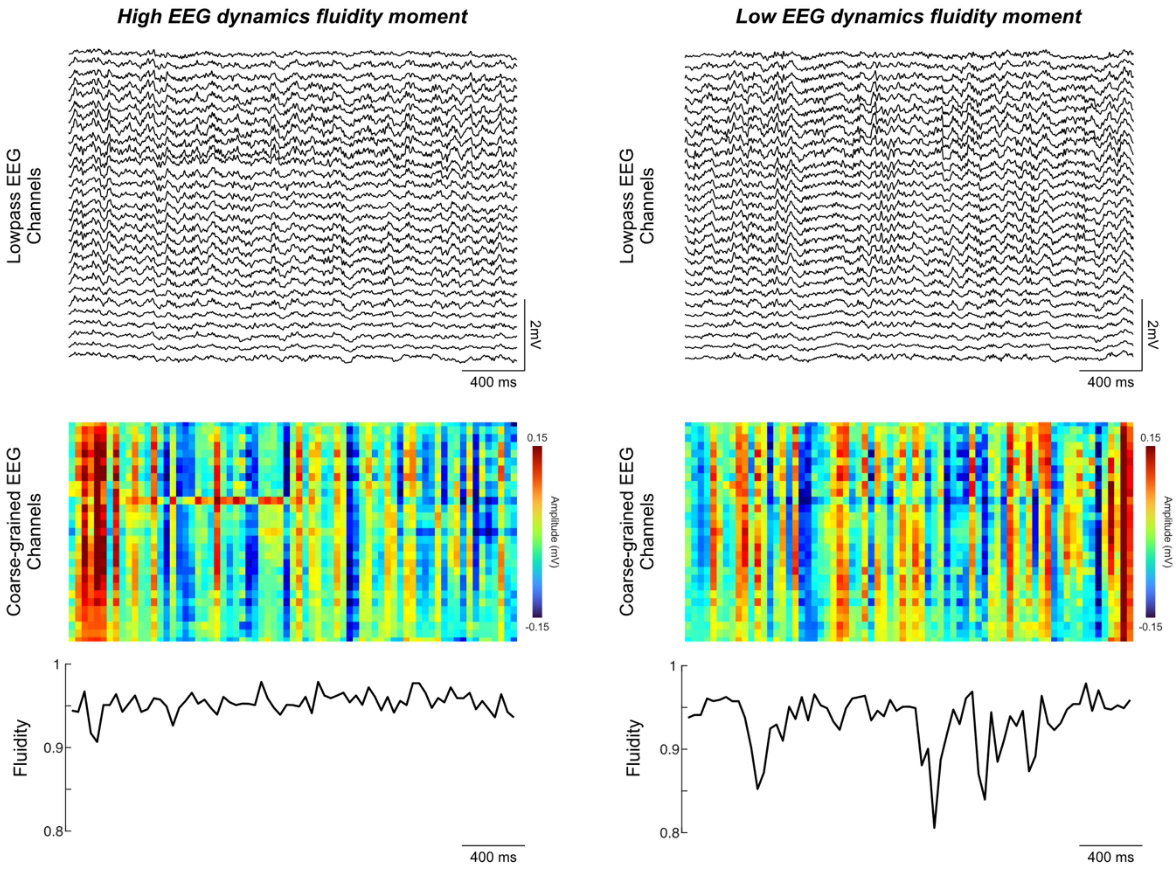

Appendix Fig. A2. Exemple of EEG dynamics fluidity. Lowpass EEG (*Top*), 40 ms coarsegrained EEG (*Middle*) and related EEG dynamics fluidty (*Bottom*) of a High fluidity (*Left*) and a Low (*Fluidity*) moment of a dKI mouse recording.

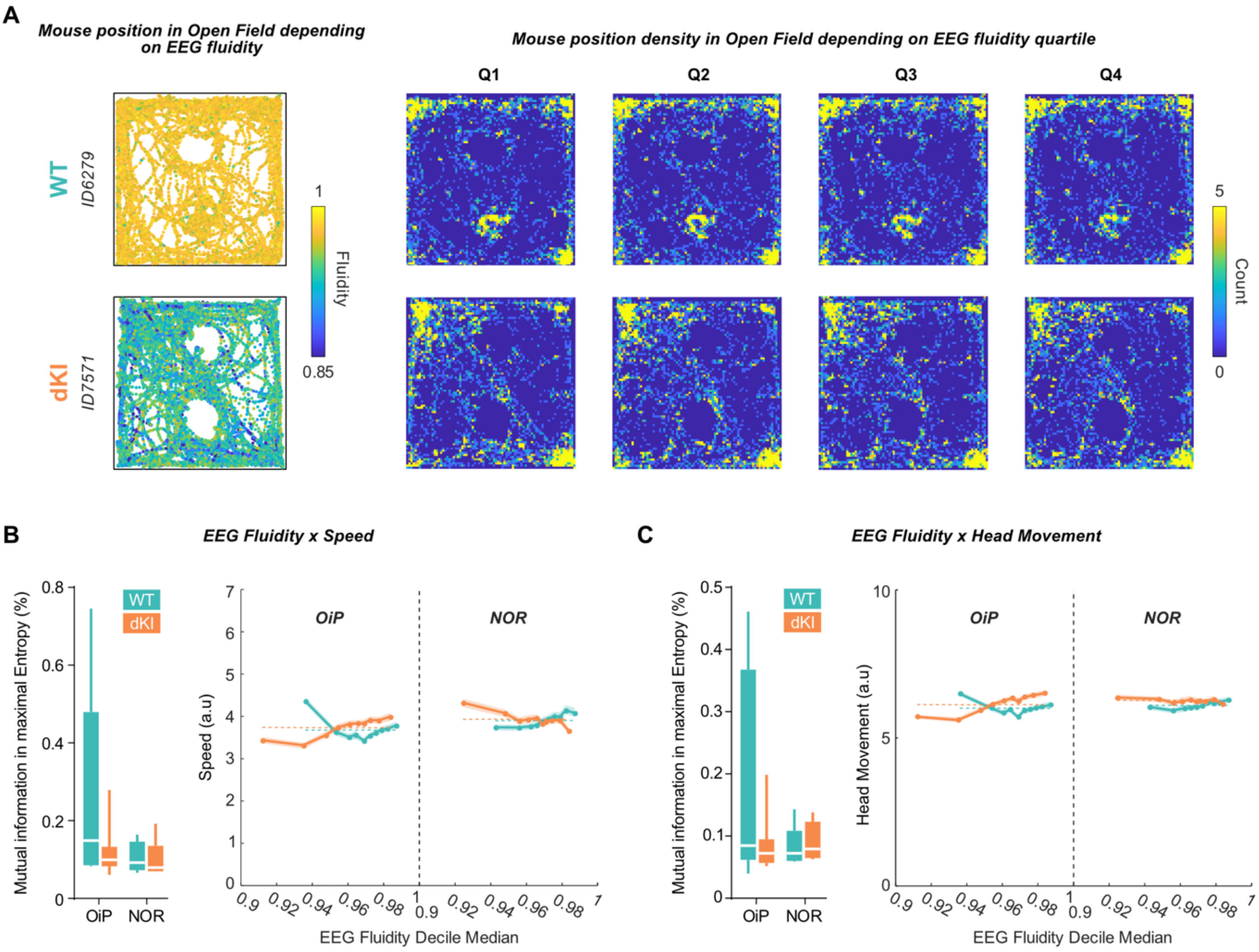

Appendix Fig. A3. EEG Fluidity do not show strong relationship with behavior. (A) Animal position in the Open Field during the performance of the OiP task in one WT (*Top*) and one dKI (*Bottom*) mouse color coded by the EEG fluidity (*Left*). Density map of the position of the animal for each quartile of EEG distribution (*Right*). All quartiles of EEG fluidity distributions are expressed in same places, showing no relationship between EEG fluidity and position in the Open Field. (B,C) Relationship between EEG fluidity and Speed of the animal (B), and between EEG fluidity and Head Movements (C). No difference in Mutual information was observed between genotype and task and value of mutual information were overall small as representing around 0.1% of the maximal entropy (*Right*). Box ranges from 25 to 75 percentile and whiskers for minimum to maximum values, median is represented by white line. *Left*, Median value of speed or Head movements for each decile of EEG fluidity reveal significant relationship between speed or head movements and Fluidity for both genotypes and task, however, given that the spread of speed and head movements values in this relation represent an extremely small portion of their distribution, no real relationship can be interpreted between these variables. Data are mean ± s.e.m, dotted line represent the bootstrap chance level.

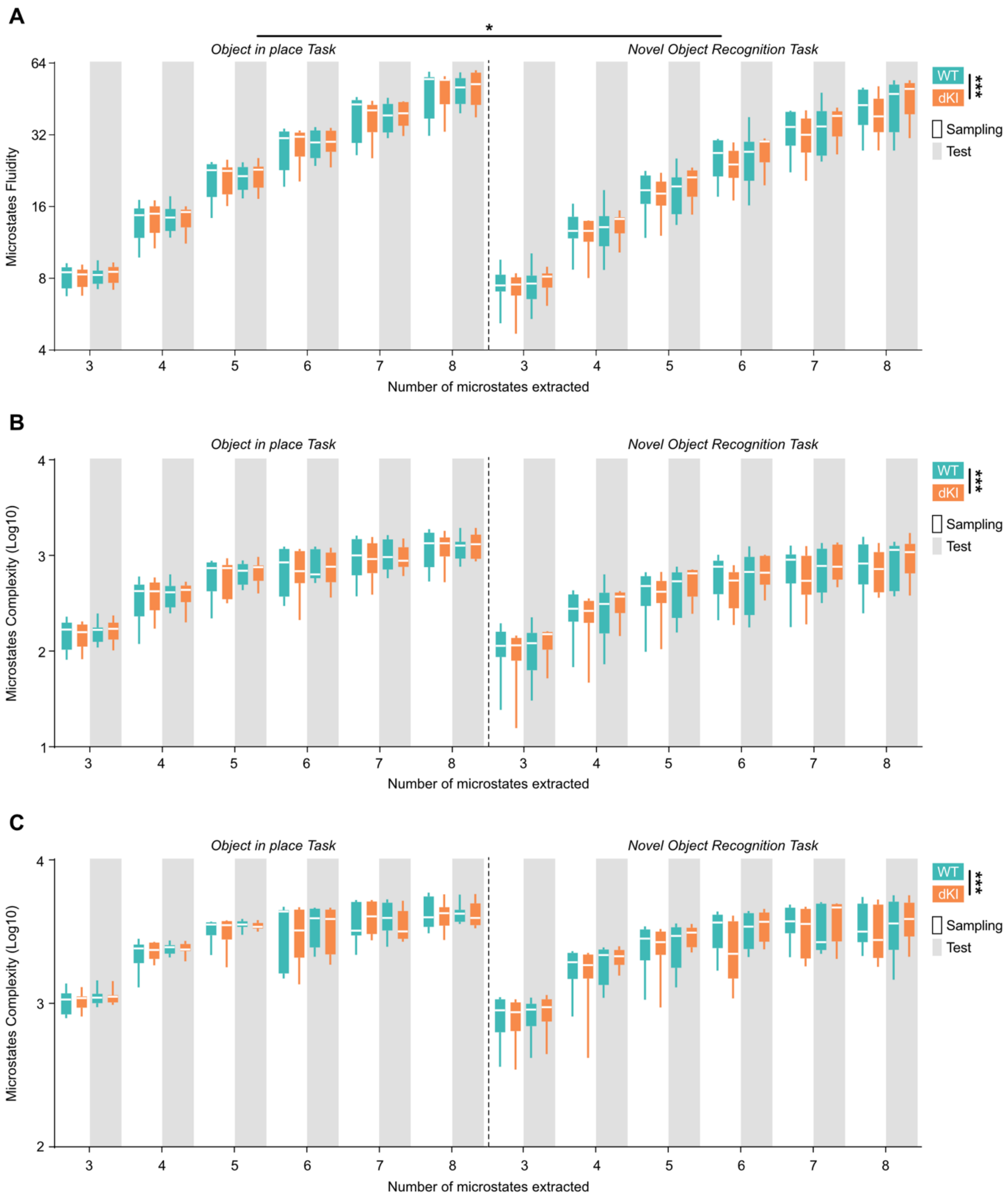

Appendix Fig. A4. Microstates dynamics and complexity alteration are independent from the number of microstates extracted and not only explained by microstates repetition. Box ranges from 25 to 75 percentile and whiskers for minimum to maximum values, median is represented by white line. (A) Microstate fluidity during sampling (no background) and test (grey background) phase of OiP and NOR tasks for WT (blue) and dKI (orange) mice (n=8 per group) across 3 to 8 microstates extracted. Four-way ANOVA (factor: Genotype, Task, Phase, Clusters; *: p<0.05 **: p<0.01 ***: p<0.001) showed

significant Genotype effect ($F(1,336) = 41.884$; $p < 0.001$) indicating a lower fluidity in dKI mice. This effect showed no interactions with Task or Phase. (B) Microstate sequence complexity during sampling (no background) and test (grey background) phase of OiP and NOR tasks for WT (blue) and dKI (orange) mice (n=8 per group) across 3 to 8 microstates extracted. Four-way ANOVA (factor: Genotype, Task, Phase, Clusters; *: $p<0.05$ **: $p<0.01$ ***: $p<0.001$) showed significant Genotype effect ($F(1,336) = 40.185$; $p < 0.001$) indicating a lower complexity in dKI mice. This effect showed no interactions with Task or Phase. (C) Repetition free microstate sequence complexity during sampling (no background) and test (grey background) phase of OiP and NOR tasks for WT (blue) and dKI (orange) mice (n=8 per group) across 3 to 8 microstates extracted. Four-way ANOVA (factor: Genotype, Task, Phase, Clusters; *: $p<0.05$ **: $p<0.01$ ***: $p<0.001$) showed significant Genotype effect ($F(1,336) = 22.701$; $p < 0.001$) indicating a lower complexity in dKI mice. This effect showed no interactions with Task or Phase.

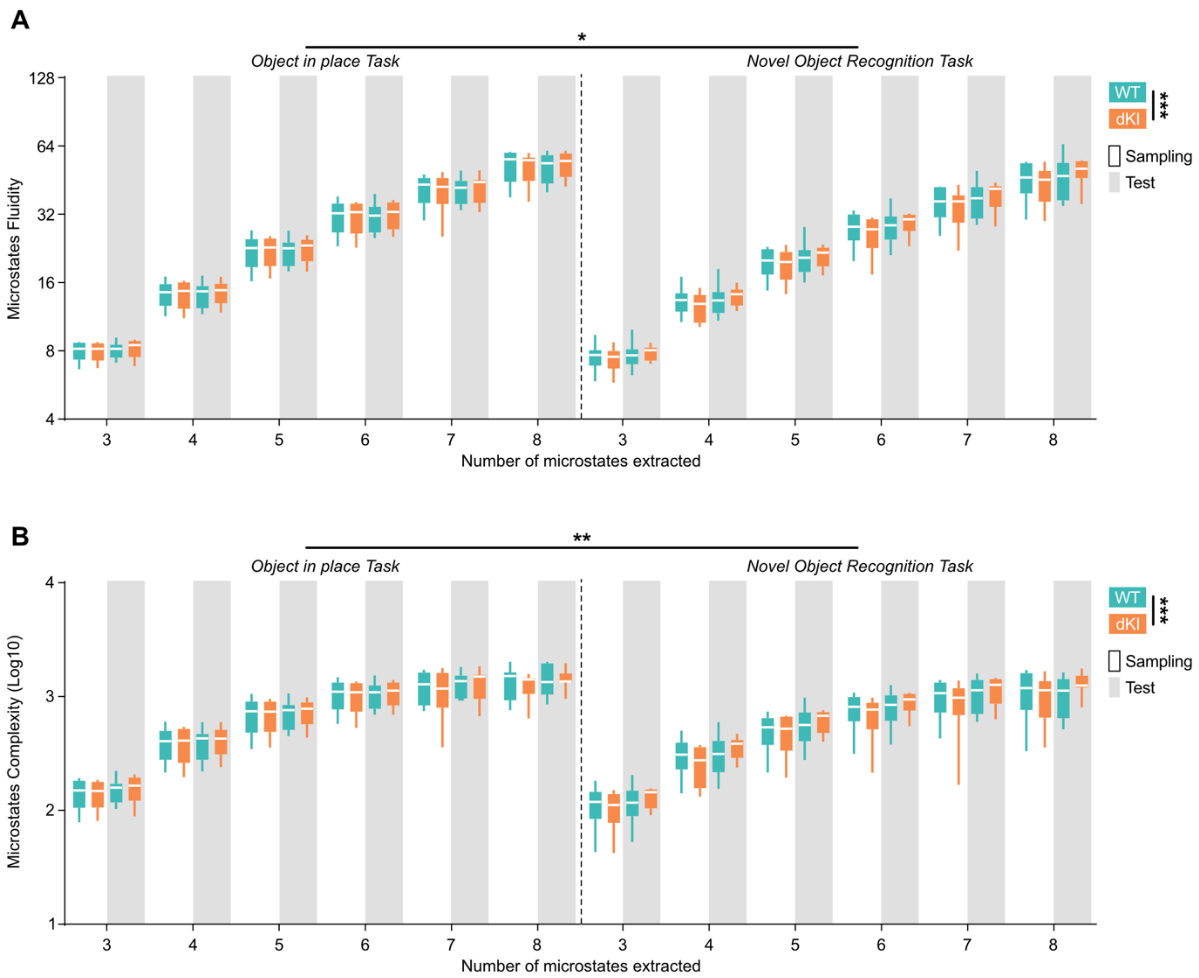

Appendix Fig. A5. Microstates sequence dynamics and complexity are altered in dKI mice and rescued by vGENUS when using Global Field Power microstates extraction method. Box ranges from 25 to 75 percentile and whiskers for minimum to maximum values, median is represented by white line. (A) Microstate fluidity during sampling (no background) and test (grey background) phase of OiP and NOR tasks for WT (blue) and dKI (orange) mice (n=8 per group) across 3 to 8 microstates extracted. Four-way ANOVA (factor: Genotype, Task, Phase, Clusters; *: $p<0.05$ **: $p<0.01$ ***: $p<0.001$) showed significant Genotype effect ($F(1,336) = 33.948$; $p < 0.001$) indicating a lower fluidity in dKI mice. This effect showed no interactions with Task or Phase. (B) Microstate sequence complexity during sampling (no background) and test (grey background) phase of OiP and NOR tasks for WT (blue) and dKI (orange) mice (n=8 per group) across 3 to 8 microstates extracted. Four-way ANOVA (factor: Genotype, Task, Phase, Clusters; *: $p<0.05$ **: $p<0.01$ ***: $p<0.001$) showed significant Genotype effect ($F(1,336) = 39.279$; $p < 0.001$) indicating a lower complexity in dKI mice. This effect showed no interactions with Task or Phase.

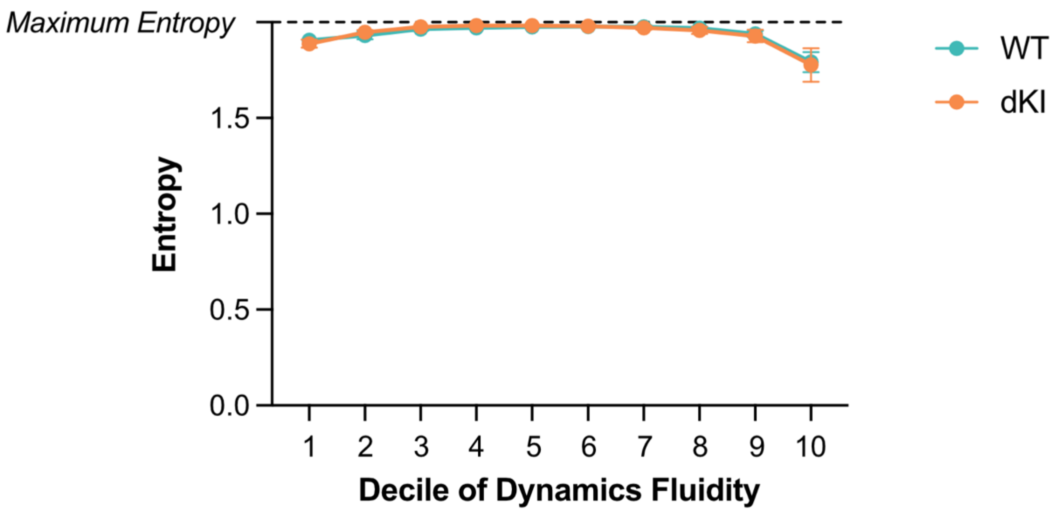

Appendix Fig. A6. EEG Dynamics fluidity drops are not linked to any specific microstates. Entropy of 4-cluster microstate sequences for each decile of EEG dynamics fluidity. For each decile, entropy values are near maximum entropy, meaning that each decile of EEG dynamics fluidity show no link with any microstates. Data are presented as mean ± s.e.m over animals.

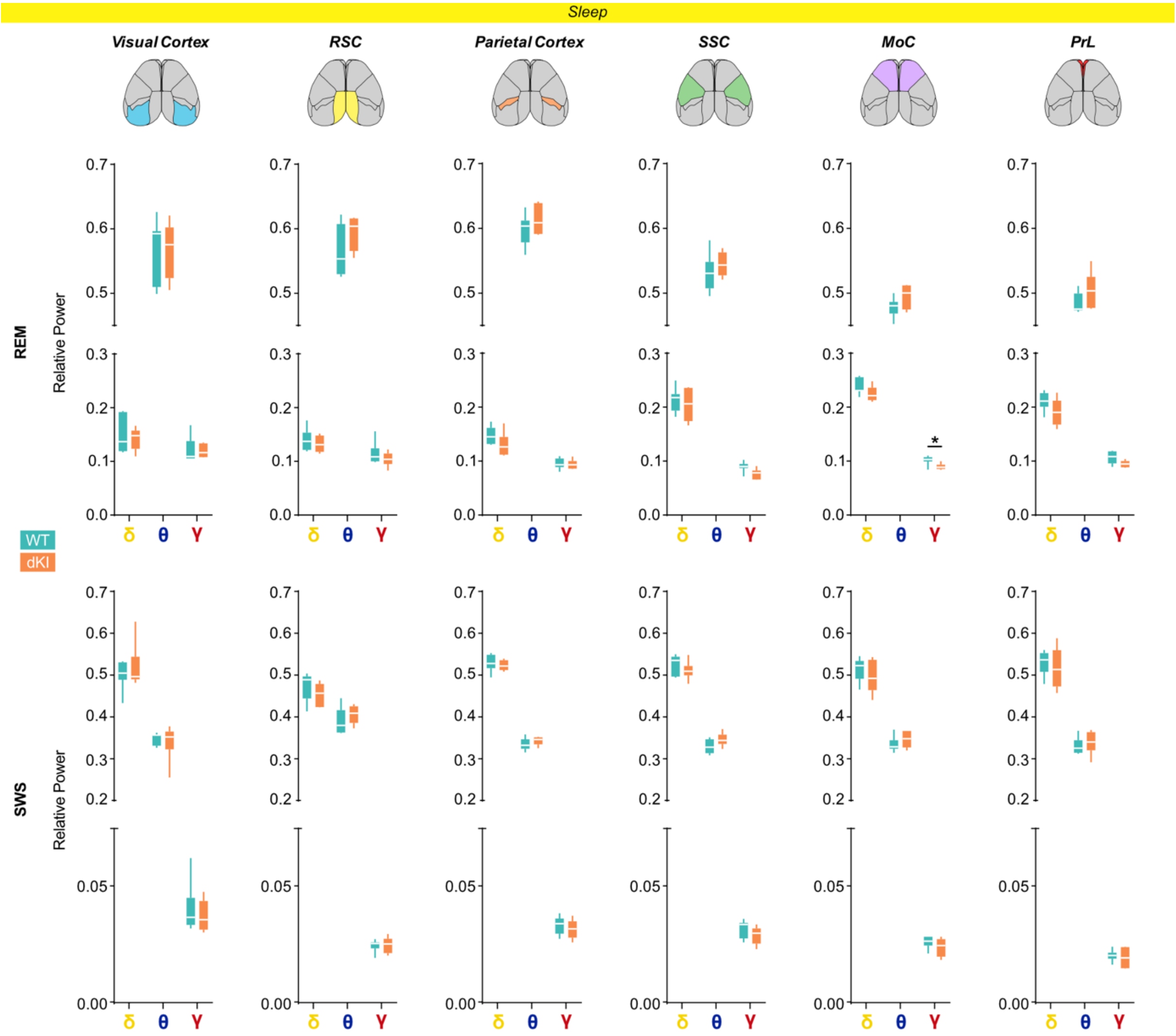

Appendix Fig. A7. dKI mice show no strong spectral power alterations during sleep. Relative power for Delta (yellow), Theta (blue) and Gamma (red) band during REM sleep (*Top*) and SWS (*Bottom*) over different cortical territories. Multiple t-test between genotypes for each region and frequency bands only reveal a slight reduction in gamma power in dKI mice over MOC during REM sleep ($t_{(11)}$ = 2.956, p = 0.039).

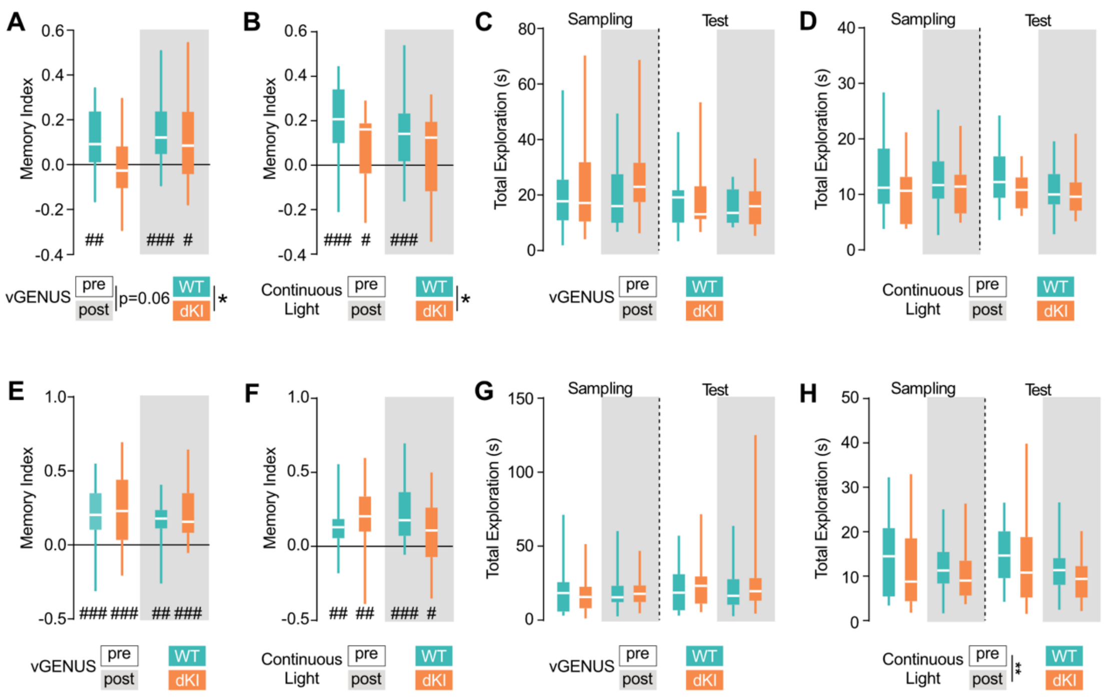

Appendix Fig. A8. vGENUS restoration of memory performances is dependant on 40Hz frequency. Box ranges from 25 to 75 percentile and whiskers for minimum to maximum values, median is represented by white line. (A) Memory index for the OiP task in WT (n=17, blue) and dKI (n=19, orange) non EEG-recorded mice pre- (no background) and post- (grey background) vGENUS. Two-way ANOVA on repeated measures showed a genotype effect (F(1,34) = 4.46, p = 0.042) but no stimulation effect (F(1,34) = 3.736, p = 0.062). In dKI mice, memory performance did not exceed chance levels pre-vGENUS but improved significantly post-vGENUS (one-sided t-test against chance, #: p < 0.05, ##: p < 0.01, ###: p < 0.001). (B) Memory index for the OiP task in WT (n=18, blue) and dKI (n=18, orange) non EEG-recorded mice pre- (no background) and post- (grey background) 2 weeks of daily control continuous light stimulation. Two-way ANOVA on repeated measures showed a genotype effect (F(1,34) = 5.38, p = 0.026) but no stimulation effect (F(1,34) = 0.4463, p = 0.509). dKI mice showed a memory index not higher from chance chance post-Continuous light (one-sided t-test against chance, #: p < 0.05, ##: p < 0.01, ###: p < 0.001). (C) Total exploration time during OiP task in WT (n=17, blue) and dKI (n=19, orange) non EEG-recorded mice pre- (no background) and post- (grey background) vGENUS. Three-way ANOVA on repeated measures showed no genotype (F(1,68) = 1.208, p = 0.276), vGENUS (F(1,68) = 0.411, p = 0.524), or phase effects (F(1,68) = 2.953, p = 0.09). (D) Total exploration time during OiP task in WT (n=18, blue) and dKI (n=18, orange) non EEG-recorded mice pre- (no background) and post- (grey background) 2 weeks of daily continuous light stimulation. Three-way ANOVA on repeated measures showed no genotype (F(1,68) = 2.901, p = 0.093), stimulation (F(1,68) = 1.324, p = 0.254), or phase effect (F(1,68) = 0.714, p = 0.401). (E) Memory index for the NOR task in WT (n=17, blue) and dKI (n=19, orange) non EEG-recorded mice pre- (no background) and post- (grey background) vGENUS. Two-way ANOVA on repeated measures showed no genotype (F(1,34) = 0.698,

$p = 0.409$) nor vGENUS effect ($F(1,34) = 0.117$, $p = 0.734$). Both WT and dKI mice memory performances were higher than chance levels pre and post-vGENUS (one-sided t-test against chance, #: $p < 0.05$, ##: $p < 0.01$, ###: $p < 0.001$). (F) Memory index for the NOR task in WT (n=18, blue) and dKI (n=18, orange) non EEG-recorded mice pre- (no background) and post- (grey background) 2 weeks of daily continuous light stimulation. Two-way ANOVA on repeated measures showed no genotype ($F(1,34) = 0.449$, $p = 0.507$) nor stimulation effect ($F(1,34) = 0.218$, $p = 0.643$). Both WT and dKI mice memory performances were higher than chance levels pre and post-Continuous light (one-sided t-test against chance, #: $p < 0.05$, ##: $p < 0.01$, ###: $p < 0.001$). (G) Total exploration time during NOR task in WT (n=17, blue) and dKI (n=19, orange) non EEG-recorded mice pre- (no background) and post- (grey background) vGENUS. Three-way ANOVA on repeated measures showed no genotype ($F(1,68) = 0.648$, $p = 0.424$), vGENUS ($F(1,68) = 0.544$, $p = 0.463$), or phase effects ($F(1,68) = 1.403$, $p = 0.240$). (H) Total exploration time during NOR task in WT (n=18, blue) and dKI (n=18, orange) non EEG-recorded mice pre- (no background) and post- (grey background) 2 weeks of daily continuous light stimulation. Three-way ANOVA on repeated measures showed no genotype ($F(1,68) = 1.367$, $p = 0.246$) or phase effect ($F(1,68) = 9.78x10-4$, $p = 0.975$) but a stimulation effect ($F(1,68) = 11.08$, $p = 0.001$) indicating a reduced exploration after 2 weeks of continuous light exposure in both genotypes.

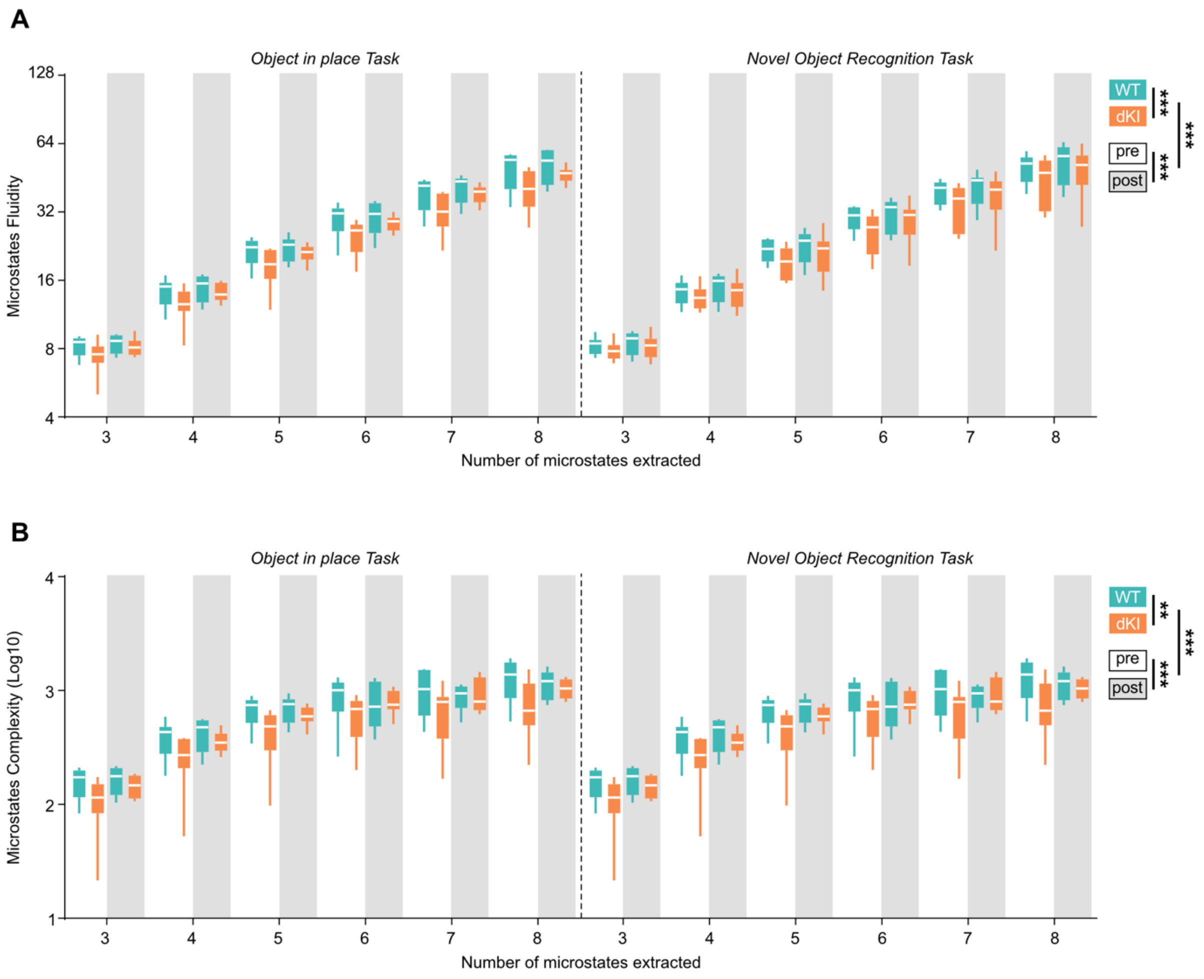

Appendix Fig. A9. Microstates dynamics and complexity restoration by vGENUS are independent from the number of microstates extracted. Box ranges from 25 to 75 percentile and whiskers for minimum to maximum values, median is represented by white line. (A) Microstate fluidity during OiP and NOR tasks for WT (blue) and dKI (orange) mice pre (no background) and post (grey background) vGENUS (n=8 per group) across 3 to 8 microstates extracted. Four-way repeated ANOVA (non –repeated factors: Genotype, Task, Clusters; repeated factor: vGENUS; *: $p<0.05$ **: $p<0.01$ ***: $p<0.001$) showed significant interaction between Genotype and vGENUS ($F(1,168) = 12.265$; $p < 0.001$). Post hoc test showed that vGENUS increased fluidity in WT ($t(168) = -3.09$; $p = 0.014$) and in dKI mice ($t(168) = -8.05$; $p <0.001$) bringing a fluidity lower than WT ($t(168) = 5.23$; $p < 0.001$) to a fluidity almost similar to WT level ($t(168) = 2.69$, $p = 0.048$). (B) Microstate sequence complexity during OiP and NOR tasks for WT (blue) and dKI (orange) mice pre (no background) and post (grey background) vGENUS (n=8 per group) across 3 to 8 microstates extracted. Four-way repeated ANOVA (non –repeated factors: Genotype, Task, Clusters; repeated factor: vGENUS; *: $p<0.05$ **: $p<0.01$ ***: $p<0.001$) showed significant interaction between Genotype and vGENUS ($F(1,168) = 15.865$; $p < 0.001$). Post hoc test showed that vGENUS increased fluidity in WT ($t(168) = -5.144$; $p < 0.001$) and in dKI mice ($t(168) = -4.869$; $p <0.001$) bringing a complexity lower than WT ($t(168) = 5.443$; $p < 0.001$) to a complexity non different to WT level ($t(168) = 2.153$, $p = 0.196$).

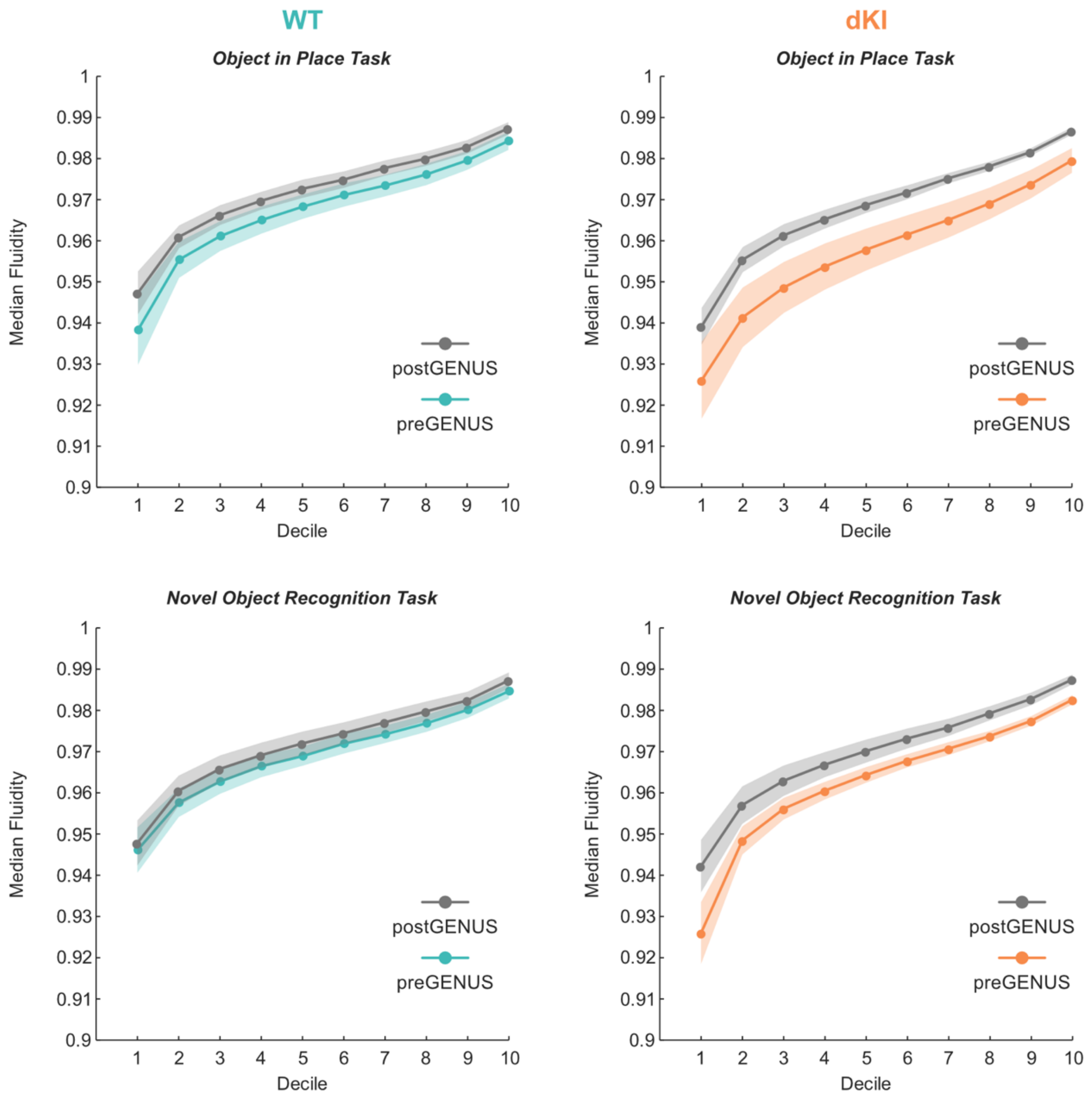

Appendix Fig. A10. vGENUS increase EEG fluidity in dKI mice by shifting the whole EEG fluidity distribution. Median EEG Fluidity value per decile for WT (n = 8, blue, *Left*) and dKI (n = 8, orange, *Right*) mice during the OiP (*Top*) and NOR (*Bottom*) tasks, before (colored) and after (gray) vGENUS protocol. Data are mean ± s.e.m.

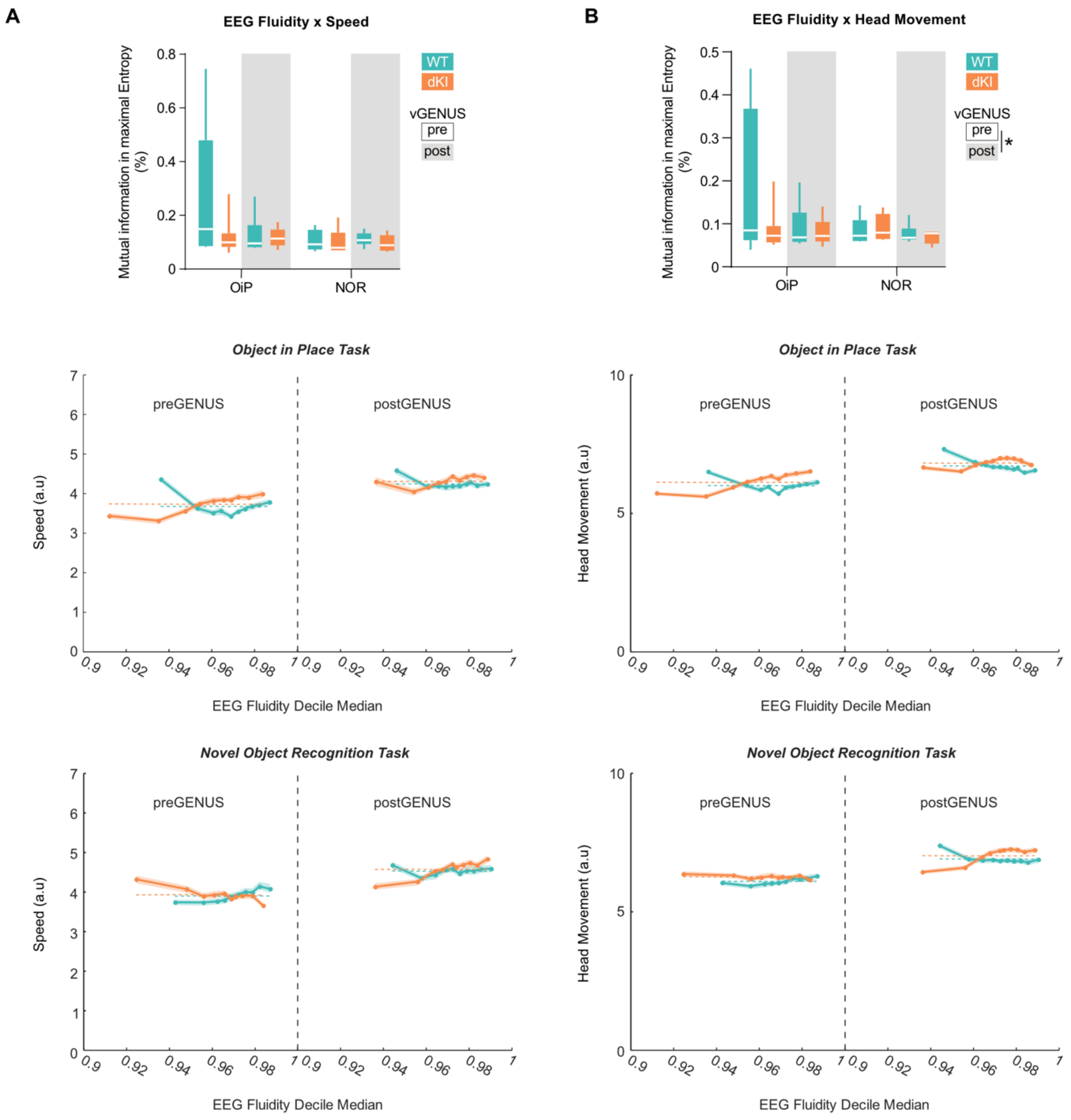

Appendix Fig. A11. vGENUS showed no clear effects on the relationship between EEG fluidity and behavior. (A, B) Relationship between EEG fluidity and Speed of the animal (A), and between EEG fluidity and Head Movements (B) before and after vGENUS. (A) No differences in EEG fluidity x speed Mutual information were observed between genotype, task and before (no background) or after (gray background) vGENUS, values of mutual information were overall small as representing around 0.1% of the maximal entropy indicating no clear relationship (*Top*). Box ranges from 25 to 75 percentile and whiskers for minimum to maximum values, median is represented by white line. Median value of speed for each decile of EEG fluidity before and after vGENUS for the OiP (*Middle*) and NOR (*Bottom*) tasks. While significant trends are observed, the spread of values is minimal, suggesting no meaningful relationship for both genotypes, both tasks and both before and after vGENUS. Data are mean ± s.e.m, dotted line represent the bootstrap chance level. (B) Three-way repeated ANOVA (non –repeated

factors: Genotype, Task; repeated factor: vGENUS; *: p<0.05 **: p<0.01 ***: p<0.001) showed no differences in EEG Fluidity x head movements Mutual information between genotype and task but a vGENUS effect was observed between before (no background) and after (gray background) vGENUS ($F_{(1, 28)}$ = 4.328, p = 0.0468) without significant post-hoc test. However, values of mutual information were overall small as representing around 0.1% of the maximal entropy indicating no clear relationship (*Top*). Box ranges from 25 to 75 percentile and whiskers for minimum to maximum values, median is represented by white line. Median value of head movement for each decile of EEG fluidity before and after vGENUS for the OiP (*Middle*) and NOR (*Bottom*) tasks. While significant trends are observed, the spread of values is minimal, suggesting no meaningful relationship for both genotypes, both tasks and both before and after vGENUS. Data are mean ± s.e.m, dotted line represent the bootstrap chance level.

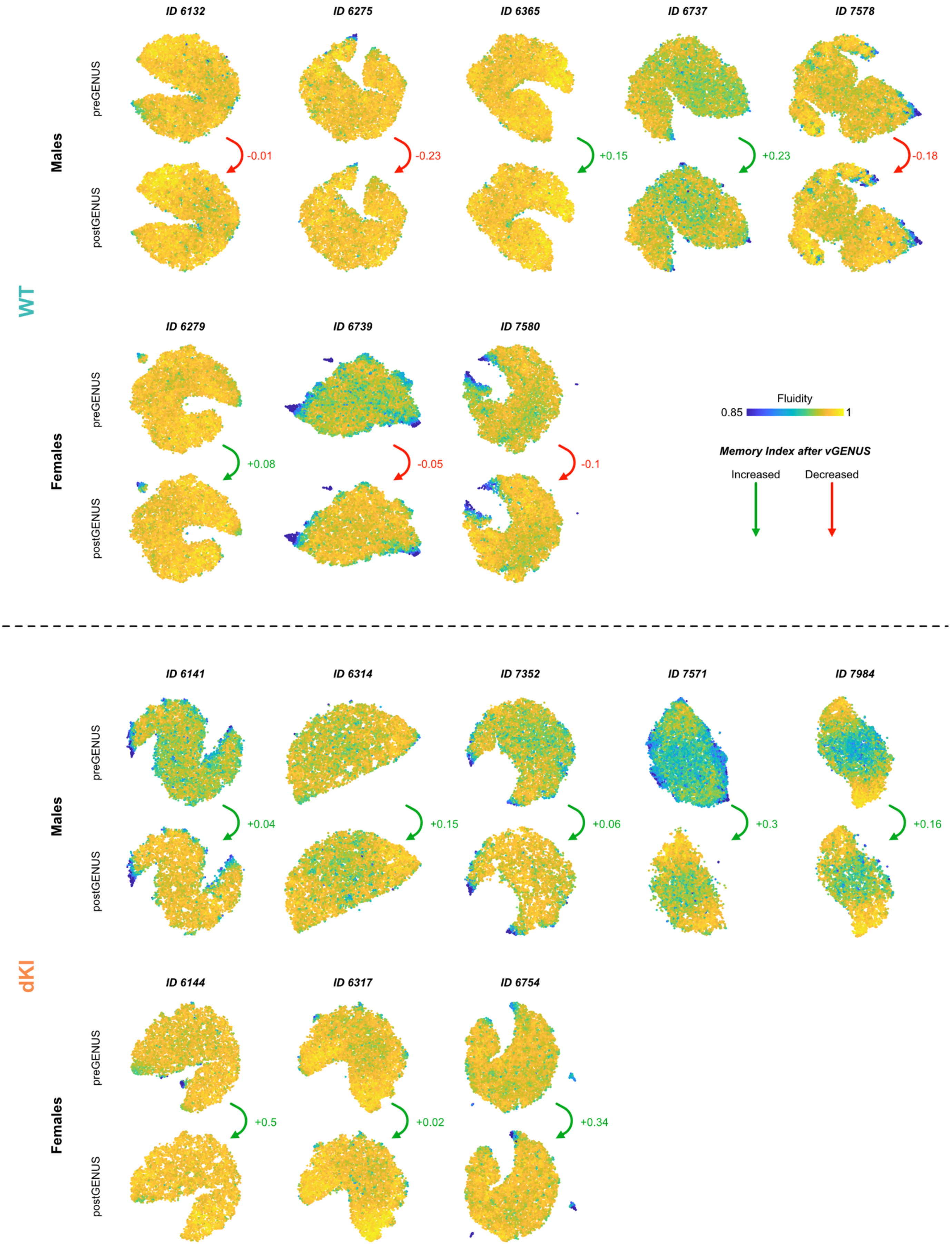

WT
Males
Females
preGENUS
postGENUS
ID 6132
ID 6275
ID 6365
ID 6737
ID 7578
-0.01
-0.23
+0.15
+0.23
-0.18
ID 6279
ID 6739
ID 7580
+0.08
-0.05
-0.1
Fluidity
0.85
1
Memory Index after vGENUS
Increased
Decreased
dKI
ID 6141
ID 6314
ID 7352
ID 7571
ID 7984
+0.04
+0.15
+0.06
+0.3
+0.16
ID 6144
ID 6317
ID 6754
+0.5
+0.02
+0.34

Appendix Fig. A12. vGENUS effects on individual EEG landscapes and fluidity. For each WT (n = 8, blue, *Top*) and dKI (n = 8, orange, *Bottom*) mouse, coarse-grained EEG recorded during the OiP task before and after vGENUS was projected onto the same t-SNE space. PreGENUS and postGENUS points are individually plotted and color-coded by EEG fluidity. Changes in memory index between pre- and postGENUS are indicated by green (increase) or red (decrease) arrows, along with the respective difference in memory index. Notably, in dKI mice, the postGENUS landscape appears more yellow, reflecting higher EEG fluidity compared to the preGENUS landscape.

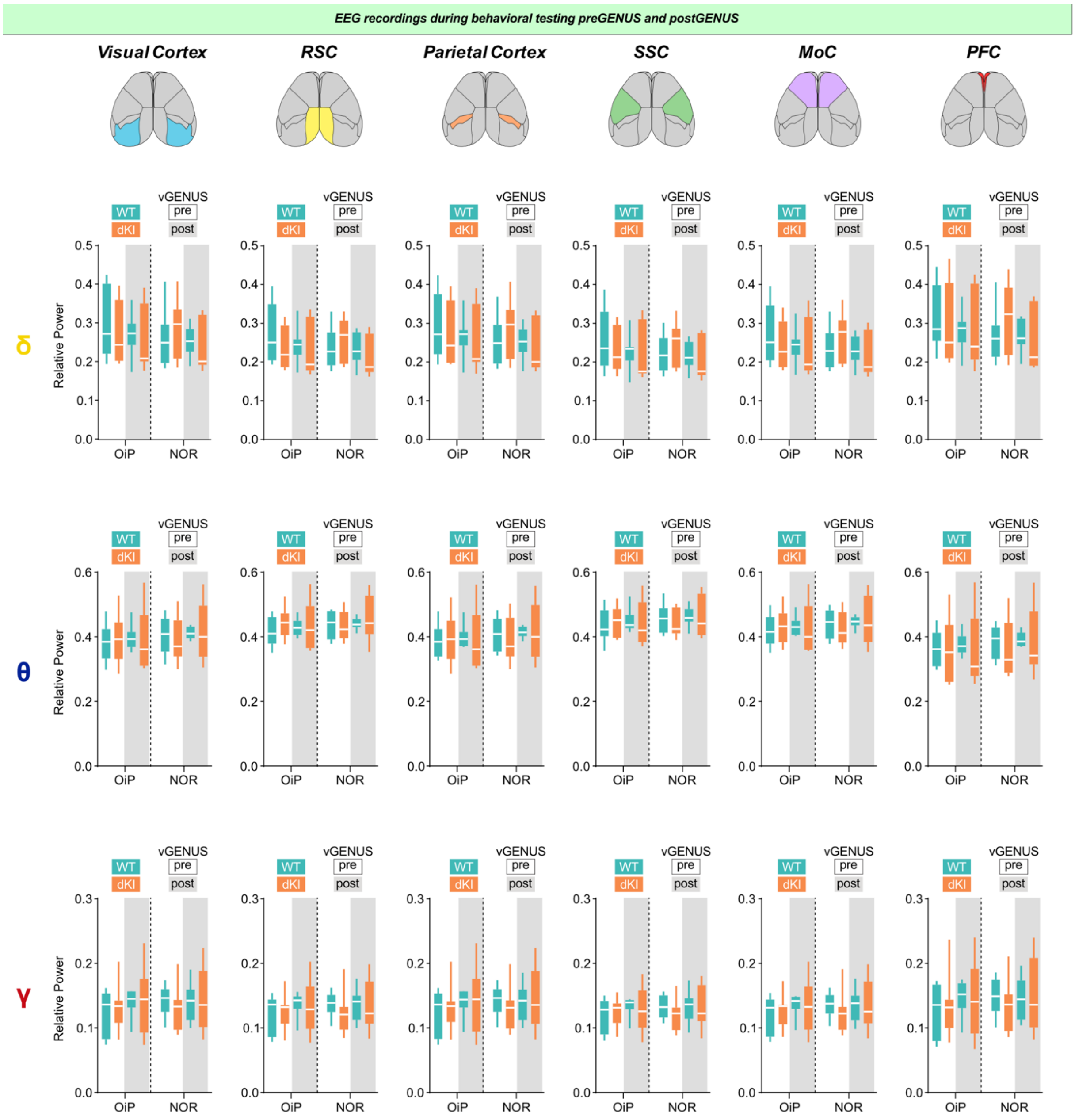

Appendix Fig. A13. vGENUS induce has no effect on EEG spectral content during performance of task. Relative power of Delta (yellow, Top), Theta (blue, Middle) and Gamma (red, Bottom) frequency band over the different cortical territories during the performance of both OiP and NOR task for WT (n = 8, blue) and dKI (n = 8, orange) mice before (no background) and after (gray background) vGENUS. Three Way ANOVA (non –repeated factors: Genotype, Task; repeated factor: vGENUS; *: $p<0.05$ **: $p<0.01$ ***: $p<0.001$) showed no effect for all frequency band and all cortical territories.

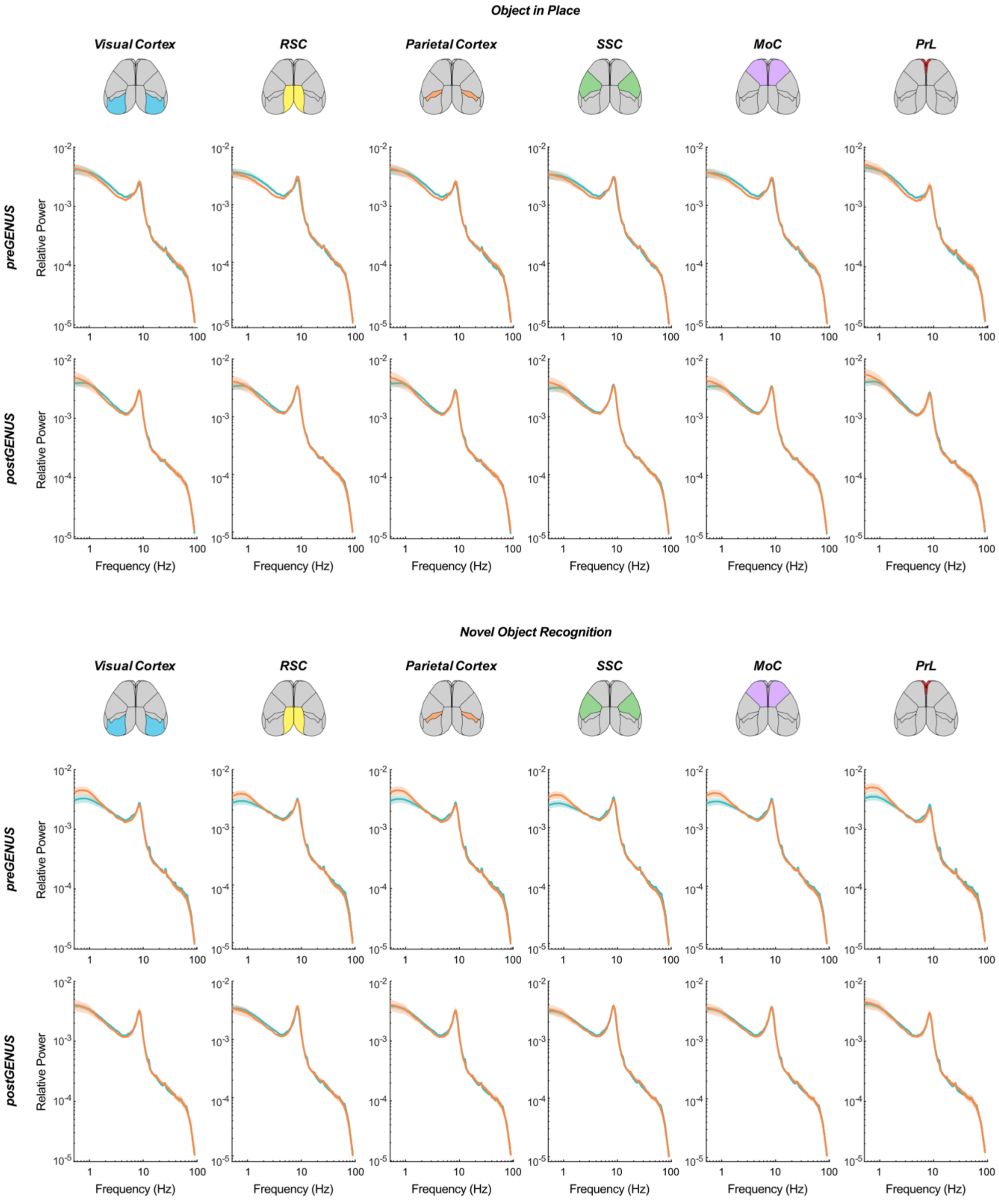

Appendix Fig. A14. Power Spectrum show no genotypes differences during behavioral task before and after vGENUS. Relative power Spectrum for WT (blue, n=8) and dKI (orange, n = 8) mice over the

different cortical territories pre and postGENUS during Object in place (*Top*) and Novel Object Recognition (*Bottom*) Task.

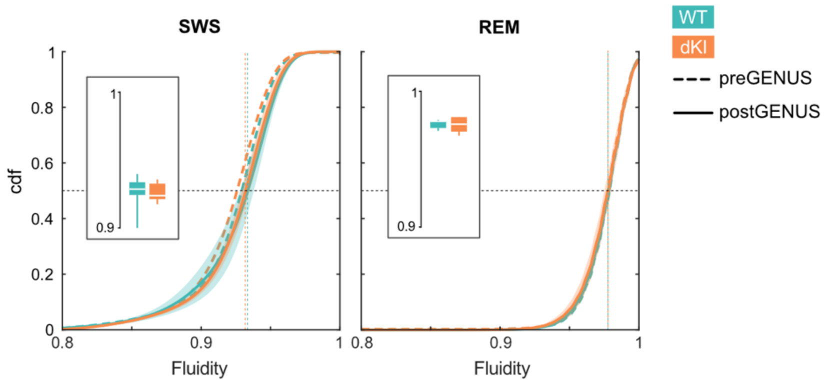

Appendix Fig. A15. vGENUS induces no change in sleep brain fluidity. Brain dynamics fluidity cumulative density distribution for WT and dKI mice show no differences in both REM and SWS after vGENUS (plain curve). No significant differences were observed for each genotype with dynamics fluidity before vGENUS (dotted curve). Data are presented as mean ± s.e.m. Dotted lines show distribution medians. Box displays individuals mean dynamics fluidity distributions.

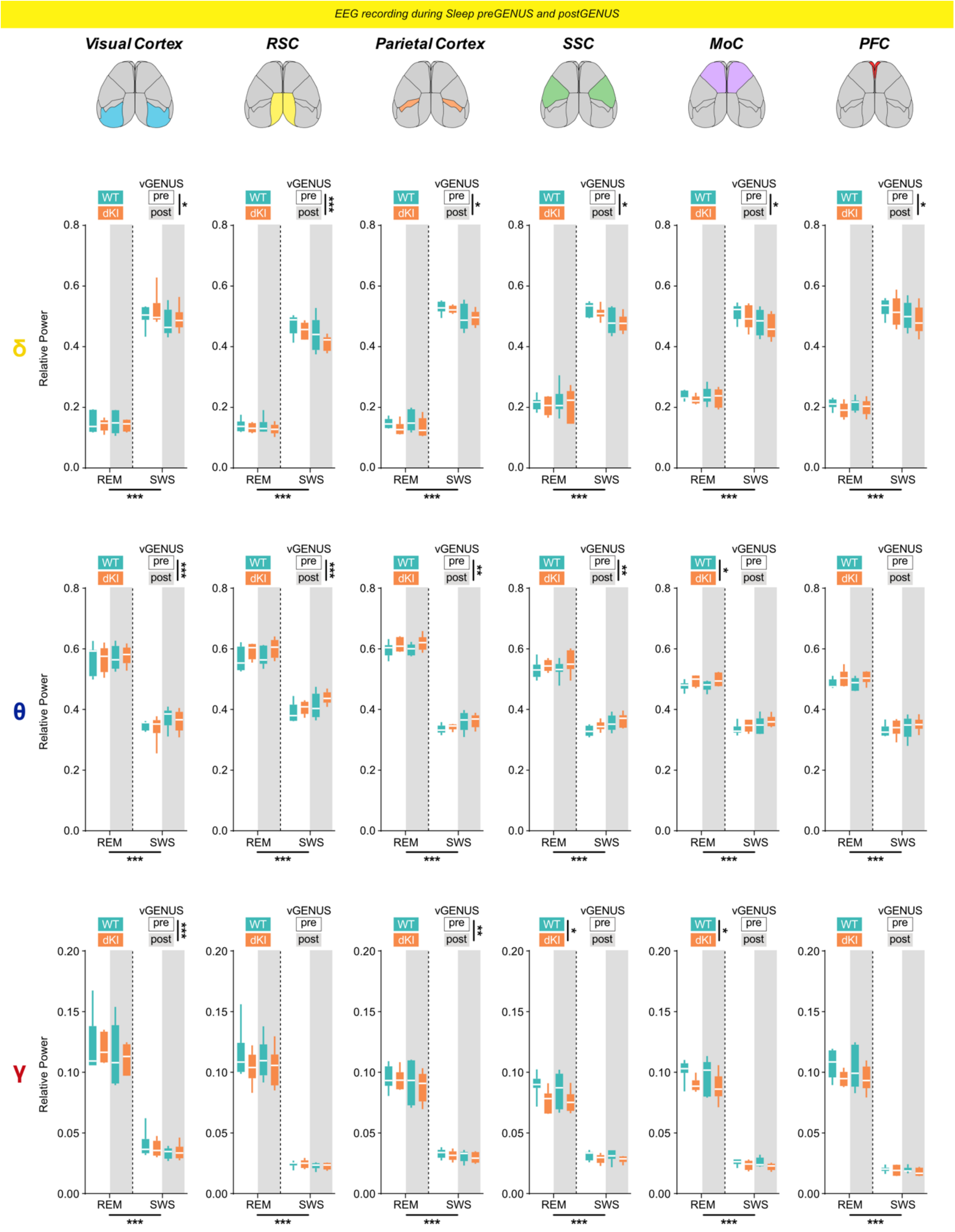

Appendix Fig. A16. vGENUS has no specific effect on dKI EEG spectral content during sleep. Relative power of Delta (yellow, Top), Theta (blue, Middle) and Gamma (red, Bottom) frequency band over the

different cortical territories during the REM and SWS for WT (n = 8, blue) and dKI (n = 8, orange) mice before (no background) and after (gray background) vGENUS. Three Way ANOVA (non –repeated factors: Genotype, Sleep Stage; repeated factor: vGENUS; *: $p<0.05$ **: $p<0.01$ ***: $p<0.001$) showed a constant Sleep stage effect logically reflecting the different spectral profile of the two sleep stage, vGENUS effect and Genotype effect were observed but interaction between Genotype and vGENUS were never observed implying the absence of a dKI specific effect of vGENUS.

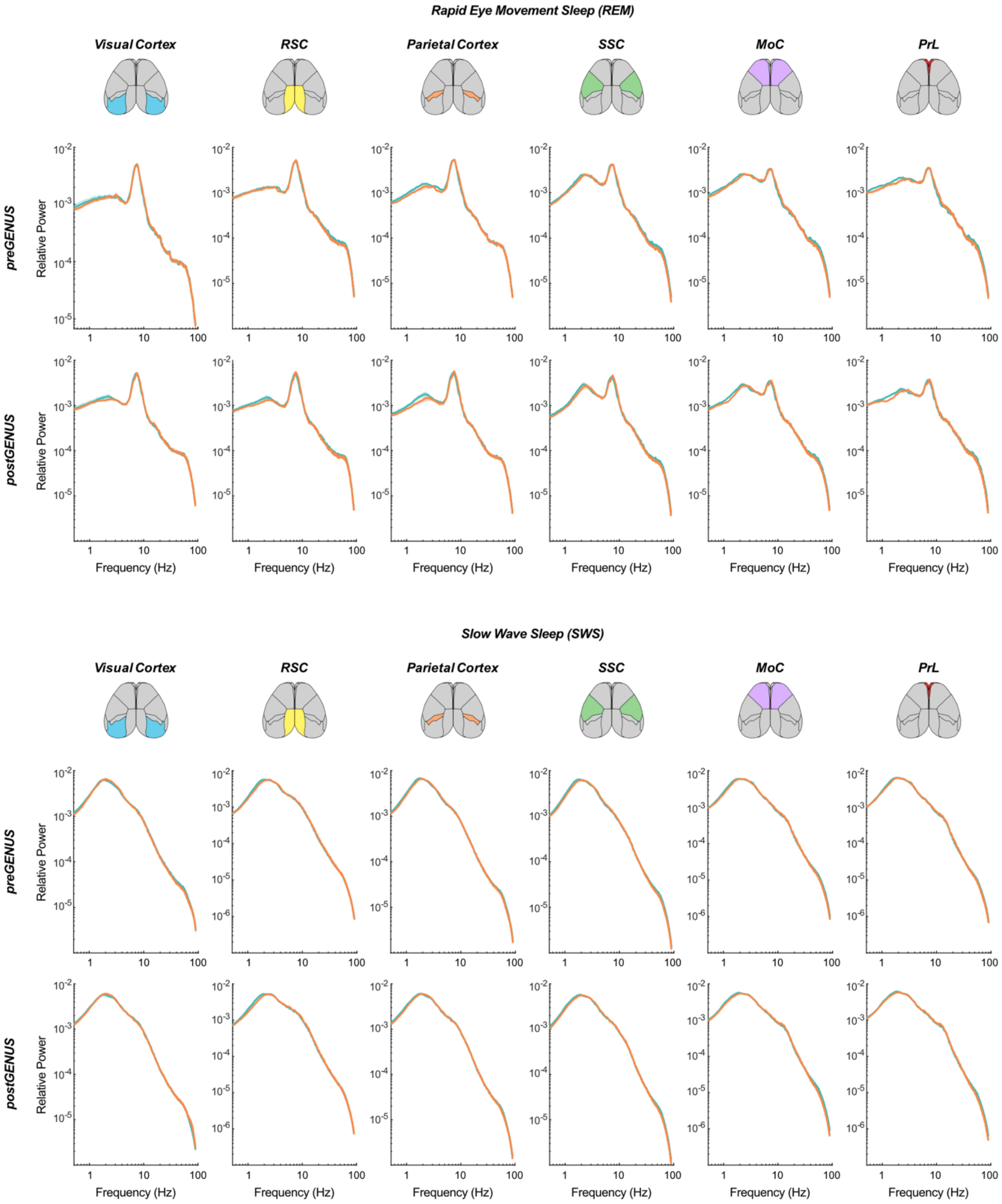

Appendix Fig. A17. vGENUS has no specific effect on dKI EEG power spectrum during sleep. Relative power Spectrum for WT (blue, n=8) and dKI (orange, n = 8) mice over the different cortical territories pre and postGENUS during REM (*Top*) and SWS (*Bottom*).
